# Impact of interspecific interactions on antimicrobial activity among soil bacteria

**DOI:** 10.3389/fmicb.2014.00567

**Published:** 2014-10-28

**Authors:** Olaf Tyc, Marlies van den Berg, Saskia Gerards, Johannes A. van Veen, Jos M. Raaijmakers, Wietse de Boer, Paolina Garbeva

**Affiliations:** ^1^Department of Microbial Ecology, Netherlands Institute of Ecology (NIOO-KNAW)Wageningen, Netherlands; ^2^Department of Soil Quality, Wageningen University and Research CentreWageningen, Netherlands

**Keywords:** soil bacteria, inter-specific interactions, high-throughput-screening, antimicrobial activity, antibiotic discovery

## Abstract

Certain bacterial species produce antimicrobial compounds only in the presence of a competing species. However, little is known on the frequency of interaction-mediated induction of antibiotic compound production in natural communities of soil bacteria. Here we developed a high-throughput method to screen for the production of antimicrobial activity by monocultures and pair-wise combinations of 146 phylogenetically different bacteria isolated from similar soil habitats. Growth responses of two human pathogenic model organisms, *Escherichia coli* WA321 and *Staphylococcus aureus* 533R4, were used to monitor antimicrobial activity. From all isolates, 33% showed antimicrobial activity only in monoculture and 42% showed activity only when tested in interactions. More bacterial isolates were active against *S. aureus* than against *E. coli*. The frequency of interaction-mediated induction of antimicrobial activity was 6% (154 interactions out of 2798) indicating that only a limited set of species combinations showed such activity. The screening revealed also interaction-mediated suppression of antimicrobial activity for 22% of all combinations tested. Whereas all patterns of antimicrobial activity (non-induced production, induced production and suppression) were seen for various bacterial classes, interaction-mediated induction of antimicrobial activity was more frequent for combinations of Flavobacteria and alpha- Proteobacteria. The results of our study give a first indication on the frequency of interference competitive interactions in natural soil bacterial communities which may forms a basis for selection of bacterial groups that are promising for the discovery of novel, cryptic antibiotics.

## Introduction

Production of antimicrobial compounds is an important strategy to increase competitiveness of soil bacteria. Soil is a heterogeneous, nutrient-poor and harsh environment harboring a huge diversity of bacteria (Gans et al., 2005; Uroz et al., 2010). There is also considerable functional redundancy as many soil bacterial species can use similar substrates as an energy source for growth and persistence (Yin et al., 2000; Strickland et al., 2009). Therefore, inter-specific competition for nutrient resources is a major type of interaction in soil bacterial communities (Demoling et al., 2007; Rousk and Baath, 2007; Rousk et al., 2009). An important strategy in interspecific interactions, known as interference competition, is the production of growth inhibitory secondary metabolites (e.g., antibiotics, toxins, biosurfactants, volatiles and others) that can suppress or kill microbial opponents (Hibbing et al., 2010; Cornforth and Foster, 2013). Although the production of antimicrobial compounds could inhibit the growth of bacterial strains competing for resources, in some cases the produced antimicrobial compounds could also promote the growth of other bacteria (D'costa et al., 2006; Dantas et al., 2008), act as signaling molecules (Linares et al., 2006; Romero et al., 2011) or modulate bacterial gene expression in sub inhibitory concentrations (Goh et al., 2002).

Whole genome sequencing has revealed that many soil microorganisms possess so-called cryptic gene clusters encoding for putative new secondary metabolites that are not produced during common *in vitro* conditions (Ikeda et al., 2003; Scherlach and Hertweck, 2009; Chiang et al., 2011; Saleh et al., 2012). In nature, however, antibiotics may be produced after perception of specific environmental signals (stress/nutrient signals) or signals from neighboring microorganisms (competitor sensing) (Firn and Jones, 2003; Cornforth and Foster, 2013; Zhu, 2014). Indeed, several studies have indicated that antibiotic production in soil bacteria can be induced when they are confronted with other bacterial species (Slattery et al., 2001; Lyon and Muir, 2003; Maurhofer et al., 2004; De Boer et al., 2007b; Seyedsayamdost et al., 2012). We hypothesize that competitor induced (facultative) rather than constitutive antibiotic production represents a key strategy in interference competition that is cost-effective and/or may reduce selection of antibiotic-resistant competitors (Garbeva et al., 2011b). Interaction-mediated induction of antibiotic production is also interesting from an applied perspective as it may lead to the discovery of novel antibiotics.

The aim of the current study was to obtain insight in the frequency of interaction-mediated induction of antibiotic production in natural soil bacterial communities. To this end, we screened a collection of bacterial isolates obtained from similar soil habitats. We developed and applied a high-throughput method to screen bacteria for the production of compounds that inhibit growth of Gram-positive and Gram-negative isolates that are closely related to human pathogens. By selecting these target organisms the study not only revealed information on the frequency of interaction-mediated antibiotic production, but also on specific soil bacterial genera or species that could be promising candidates for the discovery of novel antibiotics. The obtained results revealed that interactions have a major impact on antimicrobial compound production albeit with effects in both directions i.e., induction and suppression of antimicrobial activity.

## Materials and methods

### Soil bacteria and culture conditions

We selected 146 bacterial isolates from organic-poor, sandy soils under vegetation patches of sand sedge (*Carex arenaria* L.) growing in natural field sites (De Ridder-Duine et al., 2005) (Table S1). The bacterial isolates were pre-cultured from −80°C glycerol stocks on 1/10 TSBA (5.0 gL^−1^ NaCl, 1.0 gL^−1^ KH_2_PO_4_; 3 gL^−1^ Oxoid Tryptic Soy Broth; 20 gL^−1^ Merck Agar, pH 6.5) (Garbeva and De Boer, 2009) and incubated for 5–7 days at 20°C prior to screening.

### Control strains and target organisms

Reference strains that produce known antibiotics in monoculture were obtained from the DSMZ strain collection (Leibniz Institute DSMZ-German Collection of Microorganisms and Cell Cultures, Braunschweig, Germany). These reference strains were: *Streptomyces kanamyceticus* (DSM 40500), producer of kanamycin, *Streptomyces rimosus* (DSM 40260), producer of oxytetracycline and *Streptomyces nodosus* (DSM 40109) producer of amphotericin A and B. These strains were pre-cultured from −80°C glycerol stocks on GYM agar plates (4.0 gL^−1^ Glucose, 4.0 gL^−1^ BACTO™ Yeast extract, 10.0 gL^−1^Malt extract, 2.0 gL^−1^ CaCO_3_, 20 gL^−1^ Merck Agar, pH 7.2) and incubated for 7 days at 28°C before inoculation into 96-well source plates (see below). In the agar-overlay assay, two bacterial strains were selected to act as model organisms for human pathogenic bacteria: *Escherichia coli* WA321 (DSM 4509) as Gram-negative target organism and *Staphylococcus aureus* 533R4 Serovar 3 (DSM 20231) as Gram-positive target organism. The target strains were pre-cultured from −80°C glycerol stocks on Luria Bertani (LB) agar plates (10.0 gL^−1^ NaCl, 10 gL^−1^ Bacto™ Tryptone, 5 gL^−1^ Bacto™ Yeast extract, 20 gL^−1^ Merck Agar) Sambrook and Russell (2001) and incubated at 37°C for 24 h before inoculation in the antimicrobial screening assay. Characteristics of the target and the control strains are listed in Table S3.

### Preparation of omnitray™ plates

For the high-throughput interaction assay polystyrene Nunc™ OmniTray™—plates (size 128 × 86 mm; cap. 90 mL; Nunc™, Nalge Nunc International, Rochester, NY, USA Cat # 82-264728) were used. Each OmniTray™ plate was filled with 45 mL of 1/10 TSBA (2%) agar. Plates were kept in the laminar flow cabinet until the agar was completely solidified.

### Preparation of 96-well source-plates

**Figure 1:**
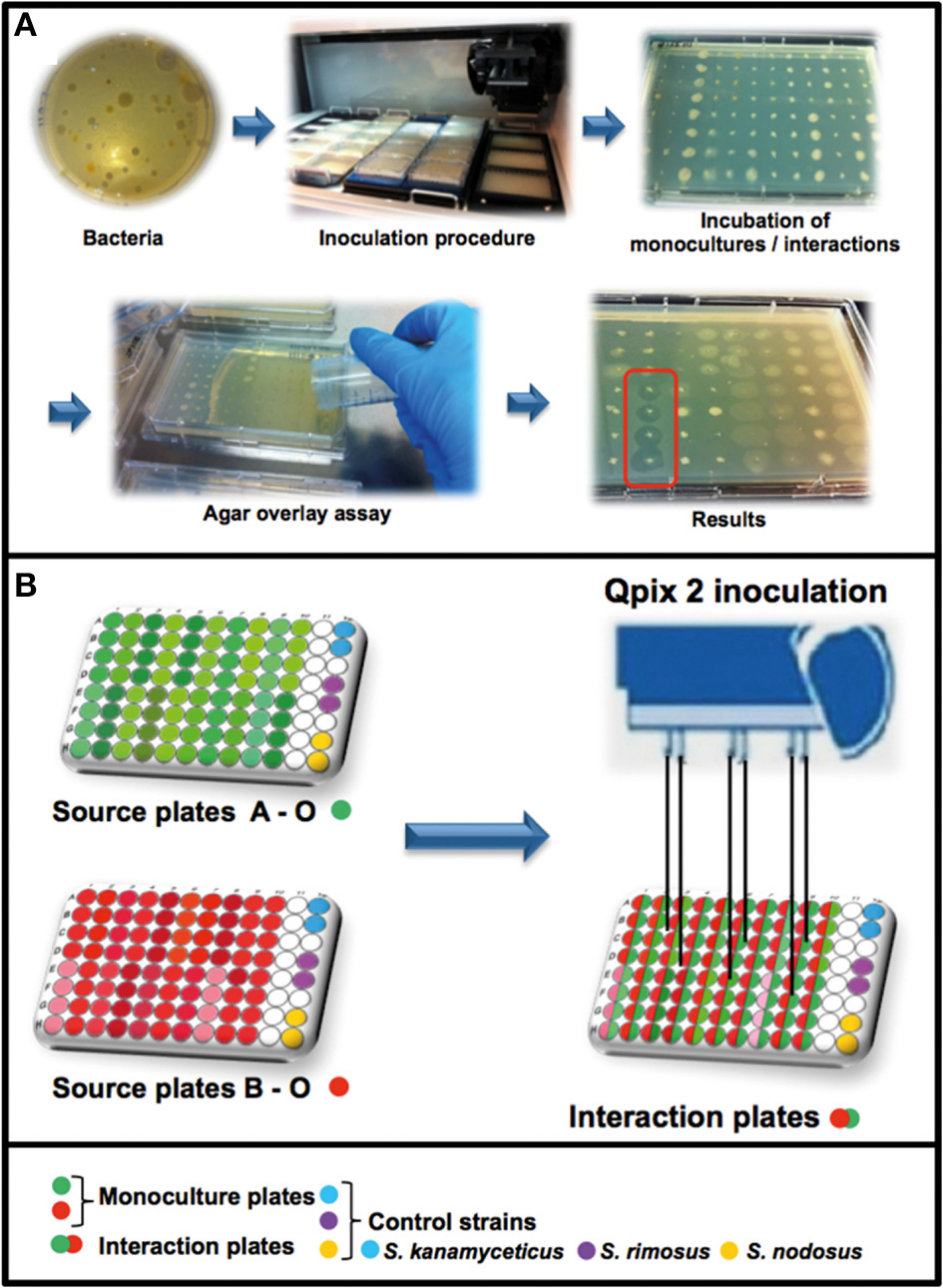
**Workflow of the high-throughput interaction assay. (A)** Overview of the antimicrobial screening: bacteria were inoculated with a Genetix Qpix 2 colony picking robot either in monoculture or in one-to-one interactions on OmniTray™ plates. For the detection of antimicrobial activity an agar overlay assay with two target organisms was performed on the fourth day of incubation. Antimicrobial activity was determined on the 5th day after overnight incubation at 37°C by screening for visible zones of inhibition (ZOI) in the upper agar layer. **(B)** Overview of the 96-well plates design and the inoculation procedure using the Genetix QPix2 colony picking robot.

96-well Microtiter plates (Greiner bio-one B.V., Alphen a/d Rijn, The Netherlands, Cat# 655180) were prepared to inoculate the selected bacterial isolates and the reference strains. Each well was filled with 150 μl liquid LB broth. Bacterial isolates were inoculated in 10 rows containing quadruplicates of each strain, the 11th row was kept empty and the 12th row was used as positive control by inoculating known antibiotic-producing *Streptomyces* strains in duplicate with one free well between each strain (Figure 1). Inoculation was done by picking cells from a single colony of each bacterial strain with a disposable inoculation loop (VWR international B.V., Amsterdam, The Netherlands Cat# 50806-404) and transferring to the designated well in the 96-well source plates. The plates were incubated for 2 days at 24°C, after which the plates were prepared for long-term storage (−80°C freezer) by adding 50 μl of 50% (v/v) glycerol to achieve a final concentration of 12.5% (v/v). In total, 15 Microtiter plates (source plates A–O) containing different compositions of monocultures of bacterial isolates were prepared for the high-throughput interaction assay.

### High-throughput interaction assay

A Genetix QPix 2 colony picking robot (Molecular Devices, UK Limited, Wokingham, United Kingdom) was used for the high-throughput interaction assay. The Genetix QPix 2 robot was mounted with a bacterial 96-pin picking head and programmed to replicate the source plates (96-well Microtiter plates) into the OmniTray™ plates (Figure 1). The source plates were replicated two times, one set of inoculated plates was removed from the robot and was used as control to estimate growth and antimicrobial activity of the monocultures. The remaining plates in the robot were used for the interaction assay by inoculating a second set of source-plates in various combinations. The second set of bacterial isolates was inoculated at the same position as the first set of bacteria, in this way the bacterial isolates had physical cell contact and could interact in one-to-one interactions (in quadruplicates). The inoculated OmniTray™ plates (monocultures and interaction plates) were incubated for 4 days at 24°C. In total, 146 bacterial isolates were combined with each other in various arrangements and tested in 2798 unique interactions for the production of antimicrobial compounds.

### Antimicrobial screening

For detection of antimicrobial activity, an agar overlay assay was performed on the 4th day of incubation (Nkanga and Hagedorn, 1978). The two target organisms *E. coli* WA321 and *S. aureus* 533R4 were grown overnight in liquid LB broth at 37°C, 220 rpm. Fresh LB- agar (1.5% Merck Agar) was prepared, cooled down to ~45°C and the target organisms were added to a final OD_600_ of 0.002 corresponding to approximately 6 × 10^∧^5 CFU/mL (*E. coli* WA321) or 4 × 10^∧^5 CFU/mL (*S. aureus* 533R4) and mixed well. A volume of 15 mL liquid LB-agar containing the target organisms was poured over the OmniTray™ plates with the empty 11th row as the start position for pouring. After solidification of the overlay agar, the OmniTray™ plates were incubated overnight at 37°C. The next day (5th day), plates were examined for visible zones of inhibition (ZOI). Monocultures or mixed-cultures of the soil bacterial isolates were scored as positive for antibiotic production if at least two out of four replicates produced zones of inhibition (Figure 1A). The majority of activity reported (>55%) involved ≥3 out of 4 replicates. For confirmation of the high-throughput screening results, several of the antibiotic-triggering/suppressing interactions were tested outside the HTS setup (Figures S7, S8).

### PCR and 16S rRNA gene sequencing

For identification of the bacterial isolates, PCRs were performed directly on colonies or with extracted genomic DNA. For genomic DNA extraction the QIAGEN QIAmp DNA Mini Kit (QIAGEN Benelux B.V., Venlo, The Netherlands cat# 51 304) was applied according to the manufacturer's manual. For the colony PCRs, a few colonies of each bacterial isolate were scraped from the plate with a disposable inoculation loop (VWR international B.V., Amsterdam, The Netherlands Cat# 50806-404) and re-suspended in 250 μl sterile MQ-water. The re-suspended bacterial cells were pulse vortexed and heated to 95°C for 5 min. Tubes were centrifuged for 3 min at 12,000×g and 1 μl supernatant from each bacterial isolate was applied in a 50 μl PCR- master mix (Promega Corp. Madison, USA cat# M7505). For 16S rRNA gene amplification, one of the two primer combinations was used: (1) forward primer pA (5′- AGA GTT TGA TCC TGG CTC AG -3′), reverse primer 1492r (5′- GRT ACC TTG TTA CGA CTT -3′), amplifying ~1492 bp from the 16S rRNA gene or (2) forward primer 27f (5′- AGA GTTT GAT CMT GGC TCAG -3′), reverse primer 1492r amplifying ~1465 bp from the 16S rRNA gene (Edwards et al., 1989; Lane, 1991) (modified). All PCR reactions were performed on a MJ Research Peltier thermal cycler 200 PCR machine (Harlow Scientific, Arlington, USA) with the following settings: initial cycle 95°C for 5 min. and 30 cycles of 94°C for 30 sec., 55°C for 30 sec. and 72°C for 1 min. After amplification, a volume of 5 μl of each PCR reaction was loaded on a 1.25 % (w/v) agarose gel and checked after electrophoresis for presence of PCR fragment. The PCR products were sent to MACROGEN (MACROGEN Europe, Amsterdam, The Netherlands) for sequencing.

### Phylogenetic analysis and sequence analysis

Obtained sequence chromatograms of the 16S rRNA gene were examined for quality and trimmed to approximately the same size (~650 bp) using 4 PEAKS V1.7.2 for MAC OS X (www.nucleobytes.com) © 2006 Mek&Tosj.com and Clustal W. The aligned 16S rRNA gene sequences were compared against those available in the NCBI database by BLASTN (blast.ncbi.nlm.nih.gov) (Altschul et al., 1997). The sequences obtained during this study are deposited in NCBI GenBank under accession numbers KJ685218–KJ685361. For two isolates, the 16S rRNA sequences were available from previous work: *P. fluorescens* (strain AD21): DQ778036, *Pedobacter* sp. (strain V48): DQ778037 (De Boer et al., 2007a).

### Network visualization of interactions

The bacterial interaction pairs that triggered or suppressed antimicrobial activity against the target organisms were visualized with Cytoscape 3.0.2 (www.cytoscape.org) for MAC OS X (Shannon et al., 2003). Interaction visualizations were performed with the following parameters: each phylogenetic class was visualized as a single node with different symbols for each phylogenetic class, the interactions between the phylogenetic classes (nodes) were visualized by links (edges) connecting each interacting phylogenetic class. Node colors were scaled to the number of interactions between the different phylogenetic classes (see Figure legends). For visualization, self-loops (interactions within the same phylogenetic class) and edges (interactions between phylogenetic classes) were bundled to single links between the respective phylogenetic classes (the darker the line the higher the number of interactions between the phylogenetic classes).

### Statistical analysis

Statistical analyses on frequencies for induction and/or suppression of antimicrobial compound production between the different Gram-groups were performed with http://math.hws.edu/javamath/ryan/ChiSquare.html using online chi square tests. Results of the chi-square test are shown in Tables S5, S6.

## Results

### Phylogeny of the tested bacterial isolates

16S rRNA gene sequence analysis revealed that the 146 bacterial isolates tested in this study belonged to 4 phyla covering 7 classes and 9 genera: Proteobacteria (14 alpha-Proteobacteria, 65 beta-Proteobacteria, 29 gamma-Proteobacteria), Bacteroidetes (19 Flavobacteria, 1 Sphingobacteria), Actinobacteria (11 Actinobacteria) and Firmicutes (7 Bacilli) (Table 1 and Table S1).

**Table 1:** **Frequencies of antimicrobial activity for the phyla included in this study**.

**Phylum/phylogenetic class**	**Total abundance**	**AM active vs. *E. coli* in monoculture**	**AM active vs. *E. coli* in interaction**	**AM active vs. *S. aureus* in monoculture**	**AM active vs. *S. aureus* in interaction**
Actinobacteria					
Actinobacteria	11	3	3	4	5
Bacteroidetes					
Flavobacteria	19	1	3	3	11
Sphingobacteria	1		1		
Firmicutes					
Bacilli	7	2	2	3	2
Proteobacteria					
a-proteobacteria	14		1	3	9
β-proteobacteria	65	17	8	26	25
γ-proteobacteria	29	2	2	12	7
(n) isolates	146	25	20	51	59

### High-throughput screening for antimicrobial activity

**Figure 2:**
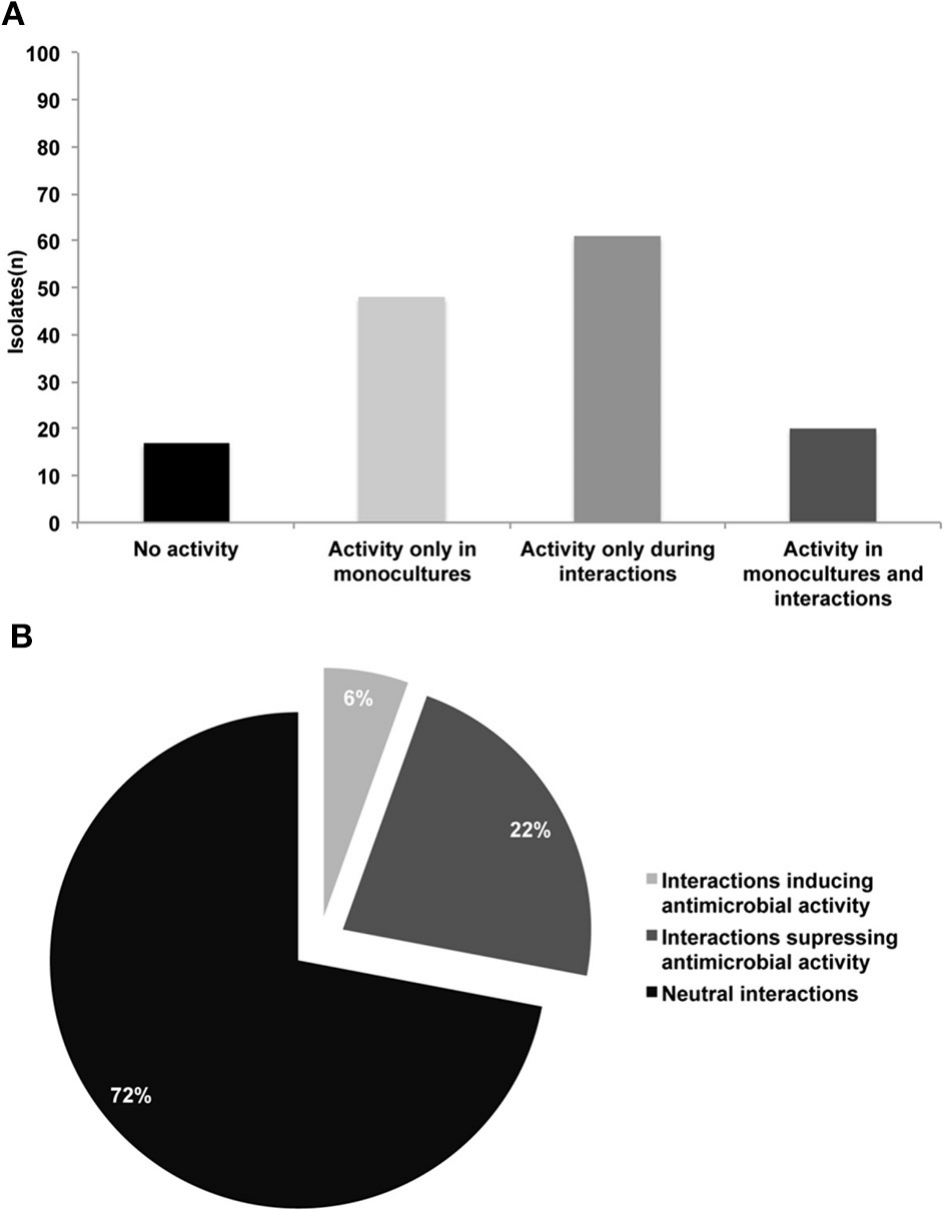
**(A)** Number of bacterial isolates exhibiting different patterns of antimicrobial activity against *E. coli* WA321 and/or *S. aureus* 533R4; in total 146 bacterial isolates were studied **(B)** Frequencies of interactions (1) inducing antimicrobial activity, (2) suppressing antimicrobial activity and (3) neutral interactions (no induction/suppression). Number of tested combinations (*n* = 2798).

**Figure 3:**
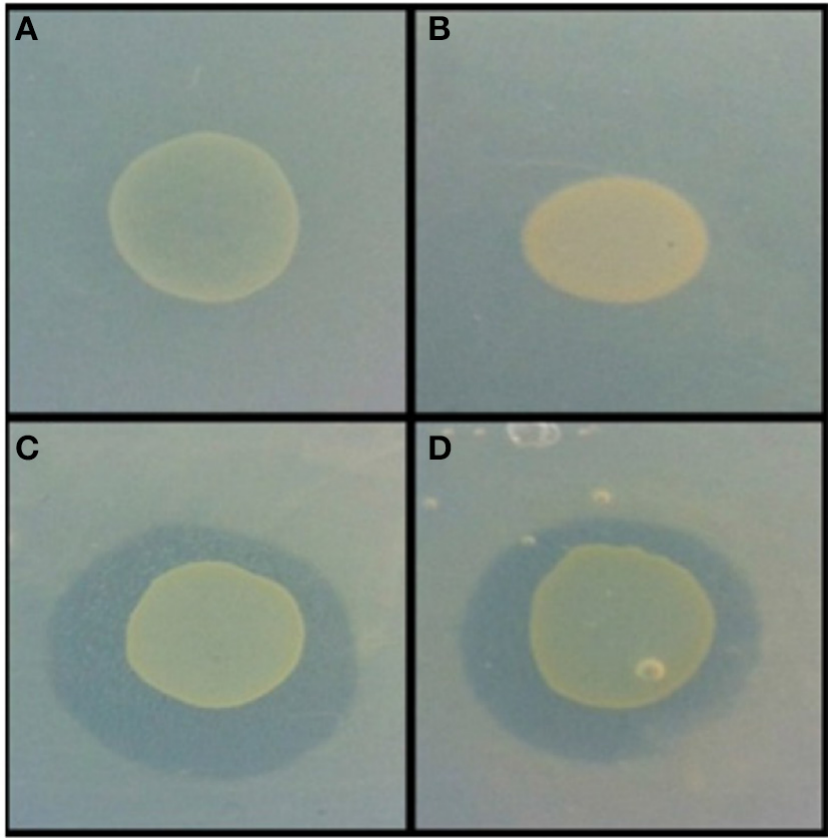
**Example of antimicrobial activity revealed via the agar overlay assay**. *Burkholderia* sp. AD24 monoculture **(A)**, *Paenibacillus* sp. AD83 monoculture **(B)**, Interaction *Burkholderia* sp. AD24 with *Paenibacillus* sp. AD83 antimicrobial activity against *S. aureus* 533R4 **(C)** and antimicrobial activity against *E. coli* WA321 **(D)**.

We developed a high-throughput assay to screen for production of antimicrobial compounds by interacting bacteria (Figure 1). In total 146 isolates were screened in monocultures and in 2798 random one-to-one interactions. For 17 isolates (11%), no activity against *E. coli* and *S. aureus* was detected not in monocultures nor in mixed cultures (Table S1 and Figure 2A). For 20 isolates (14%) antibacterial activity was observed in both monoculture and mixed cultures. For 48 isolates (33%), this was restricted to monocultures only and for 61 isolates (42%) antibacterial activity was only apparent during interactions (Figures 2A, 3). The number of isolates (110) involved in activity against the Gram-positive target strain *S. aureus* 533R4 was more than twice the number of isolates (45) with activity against the Gram-negative target strain *E. coli* WA321 (Table 1, Table S1). Despite the high number of bacterial isolates involved in antimicrobial activity in interactions, the frequency of interaction-mediated induction of antimicrobial activity was low ~6% (154 interactions out of 2798). This implies that interaction-mediated induction was only occurring in a limited number of combinations (Tables 2, 3). Most interactions (72%) did not have an effect on antimicrobial activity (induction or suppression) and about 22% of the interactions suppressed antimicrobial activity in isolates that revealed activity in monoculture (Figure 2B).

**Table 2:** **Bacterial pairs with induced antimicrobial activity against *E. coli* WA 321**.

**Phylogenetic class**	**Genus A**	**Phylogenetic class**	**Genus B**
alpha-proteobacteria	*Phyllobacterium* sp. AD152	gamma-proteobacteria	*Pseudomonas* sp. AD114
beta-proteobacteria	*Burkholderia* sp. AD24	beta-proteobacteria	*Collimonas* sp. AD68
beta-proteobacteria	*Burkholderia* sp. AD32	beta-proteobacteria	*Janthinobacterium* sp. AD80
beta-proteobacteria	*Janthinobacterium* sp. AD72	Flavobacteria	*Flavobacterium* sp. AD151
beta-proteobacteria	*Janthinobacterium* sp. AD80	gamma-proteobacteria	*Dyella* sp. AD56
beta-proteobacteria	*Janthinobacterium* sp. AD80	beta-proteobacteria	*Variovorax* sp. AD133
Actinobacteria	*Streptomyces* sp. AD108	beta-proteobacteria	*Burkholderia* sp. AD37
Actinobacteria	*Streptomyces* sp. AD108	Flavobacteria	*Flavobacterium* sp. AD47
Actinobacteria	*Streptomyces* sp. AD108	Flavobacteria	*Flavobacterium* sp. AD84
Actinobacteria	*Streptomyces* sp. AD108	Sphingobacteria	*Pedobacter* sp. V48
Actinobacteria	*Microbacterium* sp. AD141	beta-proteobacteria	*Janthinobacterium* sp. AD80
Bacilli	*Bacillus* sp. AD78	beta-proteobacteria	*Burkholderia* sp. AD11
Bacilli	*Paenibacillus* sp. AD83	beta-proteobacteria	*Burkholderia* sp. AD24
Actinobacteria	*Micrococcus* sp. AD31	Actinobacteria	*Microbacterium* sp. AD141

**Table 3:** **Bacterial pairs with induced antimicrobial activity against *S. aureus* 533R4**.

**Phylogenetic class**	**Genus A**	**Phylogenetic class**	**Genus B**
alpha-proteobacteria	*Phyllobacterium* sp. AD34	beta-proteobacteria	*Collimonas* sp. AD89
alpha-proteobacteria	*Phyllobacterium* sp. AD153	beta-proteobacteria	*Collimonas* sp. AD65
beta-proteobacteria	*Collimonas* sp. AD69	Flavobacteria	*Flavobacterium* sp. AD43
beta-proteobacteria	*Janthinobacterium* sp. AD72	gamma-proteobacteria	*Dyella* sp. AD46
beta-proteobacteria	*Janthinobacterium* sp. AD72	beta-proteobacteria	*Collimonas* sp. AD97
beta-proteobacteria	*Janthinobacterium* sp. AD72	alpha-proteobacteria	*Agrobacterium* sp. AD140
beta-proteobacteria	*Collimonas* sp. AD61	beta-proteobacteria	*Collimonas* sp. AD98
beta-proteobacteria	*Collimonas* sp. AD67	beta-proteobacteria	*Collimonas* sp. AD68
beta-proteobacteria	*Janthinobacterium* sp. AD75	beta-proteobacteria	*Burkholderia* sp. AD37
beta-proteobacteria	*Collimonas* sp. AD69	Flavobacteria	*Flavobacterium* sp. AD146
beta-proteobacteria	*Collimonas* sp. AD71	alpha-proteobacteria	*Rhizobium* sp. AD148
beta-proteobacteria	*Collimonas* sp. AD88	beta-proteobacteria	*Burkholderia* sp. AD37
beta-proteobacteria	*Collimonas* sp. AD102	Flavobacteria	*Flavobacterium* sp. AD45
beta-proteobacteria	*Collimonas* sp. AD98	Flavobacteria	*Flavobacterium* sp. AD142
beta-proteobacteria	*Burkholderia* sp. AD37	gamma-proteobacteria	*Pseudomonas* sp. AD104
beta-proteobacteria	*Collimonas* sp. AD99	beta-proteobacteria	*Burkholderia* sp. AD138
beta-proteobacteria	*Collimonas* sp. AD89	alpha-proteobacteria	*Mesorhizobium* sp. AD38
beta-proteobacteria	*Variovorax* sp. AD143	beta-proteobacteria	*Collimonas* sp. AD65
beta-proteobacteria	*Variovorax* sp. AD143	alpha-proteobacteria	*Mesorhizobium* sp. AD112
beta-proteobacteria	*Variovorax* sp. AD143	alpha-proteobacteria	*Phyllobacterium* sp. AD153
beta-proteobacteria	*Collimonas* sp. AD98	alpha-proteobacteria	*Phyllobacterium* sp. AD159
beta-proteobacteria	*Collimonas* sp. AD98	gamma-proteobacteria	*Pseudomonas* sp. AD105
beta-proteobacteria	*Collimonas* sp. AD137	gamma-proteobacteria	*Pseudomonas* sp. AD157
beta-proteobacteria	*Collimonas* sp. AD97	beta-proteobacteria	*Collimonas* sp. AD62
beta-proteobacteria	*Roseateles* sp. AD145	beta-proteobacteria	*Collimonas* sp. AD67
gamma-proteobacteria	*Pseudomonas* sp. AD124	beta-proteobacteria	*Collimonas* sp. AD65
gamma-proteobacteria	*Pseudomonas* sp. AD114	beta-proteobacteria	*Burkholderia* sp. AD18
gamma-proteobacteria	*Pseudomonas* sp. AD105	alpha-proteobacteria	*Bosea* sp. AD132
gamma-proteobacteria	*Pseudomonas* sp. AD104	alpha-proteobacteria	*Phyllobacterium* sp. AD136
gamma-proteobacteria	*Pseudomonas* sp. AD104	Flavobacteria	*Chryseobacterium* sp. AD48
Flavobacteria	*Flavobacterium* sp. AD91	beta-proteobacteria	*Variovorax* sp. AD143
Flavobacteria	*Flavobacterium* sp. AD91	alpha-proteobacteria	*Phyllobacterium* sp. AD153
Flavobacteria	*Flavobacterium* sp. AD42	Flavobacteria	*Flavobacterium* sp. AD146
Flavobacteria	*Flavobacterium* sp. AD155	beta-proteobacteria	*Collimonas* sp. AD98
Flavobacteria	*Flavobacterium* sp. AD44	beta-proteobacteria	*Collimonas* sp. AD62
Actinobacteria	*Micrococcus* sp. AD31	beta-proteobacteria	*Collimonas* sp. AD65
Actinobacteria	*Micrococcus* sp. AD31	beta-proteobacteria	*Collimonas* sp. AD69
Actinobacteria	*Micrococcus* sp. AD31	beta-proteobacteria	*Collimonas* sp. AD70
Actinobacteria	*Micrococcus* sp. AD31	Flavobacteria	*Flavobacterium* sp. AD85
Actinobacteria	*Micrococcus* sp. AD31	beta-proteobacteria	*Collimonas* sp. AD88
Actinobacteria	*Micrococcus* sp. AD31	alpha-proteobacteria	*Phyllobacterium* sp. AD136
Actinobacteria	*Micrococcus* sp. AD31	gamma-proteobacteria	*Stenotrophomonas* sp. AD147
Actinobacteria	*Micrococcus* sp. AD31	Flavobacteria	*Flavobacterium* sp. AD156
Actinobacteria	*Streptomyces* sp. AD92	beta-proteobacteria	*Collimonas* sp. AD65
Actinobacteria	*Streptomyces* sp. AD92	beta-proteobacteria	*Variovorax* sp. AD143
Actinobacteria	*Streptomyces* sp. AD92	beta-proteobacteria	*Burkholderia* sp. AD18
Actinobacteria	*Streptomyces* sp. AD92	alpha-proteobacteria	*Phyllobacterium* sp. AD153
Actinobacteria	*Tsukamurella* sp. AD106	beta-proteobacteria	*Collimonas* sp. AD89
Actinobacteria	*Tsukamurella* sp. AD106	Flavobacteria	*Chryseobacterium* sp. AD48
Actinobacteria	*Streptomyces* sp. AD108	beta-proteobacteria	*Burkholderia* sp. AD37
Actinobacteria	*Streptomyces* sp. AD108	Flavobacteria	*Chryseobacterium* sp. AD48
Actinobacteria	*Streptomyces* sp. AD108	beta-proteobacteria	*Janthinobacterium* sp. AD73
Actinobacteria	*Streptomyces* sp. AD108	beta-proteobacteria	*Janthinobacterium* sp. AD75
Actinobacteria	*Streptomyces* sp. AD108	beta-proteobacteria	*Collimonas* sp. AD88
Actinobacteria	*Streptomyces* sp. AD108	beta-proteobacteria	*Collimonas* sp. AD101
Actinobacteria	*Streptomyces* sp. AD108	gamma-proteobacteria	*Pseudomonas* sp. AD104
Actinobacteria	*Microbacterium* sp. AD141	beta-proteobacteria	*Burkholderia* sp. AD37
Bacilli	*Paenibacillus* sp. AD83	beta-proteobacteria	*Collimonas* sp. AD62
Bacilli	*Paenibacillus* sp. AD83	beta-proteobacteria	*Burkholderia* sp. AD24
Bacilli	*Paenibacillus* sp. AD116	gamma-proteobacteria	*Pseudomonas* sp. AD104
Actinobacteria	*Micrococcus* sp. AD31	Actinobacteria	*Tsukamurella* sp. AD106
Actinobacteria	*Tsukamurella* sp. AD106	Actinobacteria	*Microbacterium* sp. AD141
Actinobacteria	*Streptomyces* sp. AD108	Actinobacteria	*Microbacterium* sp. AD141

### Antimicrobial activity during interactions

#### Interaction-mediated activity against E. coli WA321

Growth of *E. coli* WA321 was inhibited by 14 pair-wise combinations involving 20 isolates that did not show antimicrobial activity in monoculture (Table 2). Some isolates were present in different combinations. For example, *Janthinobacterium* sp. AD80 and *Streptomyces* sp. AD108 were present in 4 combinations with induced activity (Table 2). Combinations inhibiting growth of *E. coli* WA321 consisted of Gram-negative/Gram-positive isolates (7 interactions) or Gram-negative/Gram-negative (6 interactions). Only in one case, a combination of two Gram-positive isolates (*Micrococcus* and *Microbacterium*) showed activity against *E. coli*.

#### Interaction-mediated activity against S. aureus 533R4

Growth of *S. aureus* 533R4 was inhibited by 63 pair-wise combinations involving 59 isolates. Several isolates were present in multiple combinations that inhibited growth of *S. aureus* (Table 3). *Burkholderia* sp. AD37, *Collimonas* sp. AD65, *Collimonas* sp. AD98, *Janthinobacterium* sp. AD72, *Micrococcus* sp. AD31, *Pseudomonas* sp. AD104, *Streptomyces* spp. AD92 and AD108, *Variovorax* sp. AD143 were all involved in more than five combinations that inhibited the growth of *S. aureus*. Most of the combinations consisted of Gram-negative/Gram-negative isolates (35 interactions) or Gram-negative/Gram-positive isolates (25 interactions). Activity against *S. aureus* was only observed 3 times for Gram-positive/Gram-positive combinations (Figure S2).

#### Interaction-mediated activity against both target organisms

Nine isolates were present in pair-wise combinations that exhibited antimicrobial activity against both target organisms (Table S1). Two combinations were inhibitory for both target organisms. These were the combinations of *Burkholderia* sp. AD24 and *Paenibacillus* sp. AD83 (Figure 3) and of *Streptomyces* sp. AD108 and *Burkholderia* sp. AD37.

#### Interactions inducing antimicrobial activity against E. coli or S. aureus

**Figure 4:**
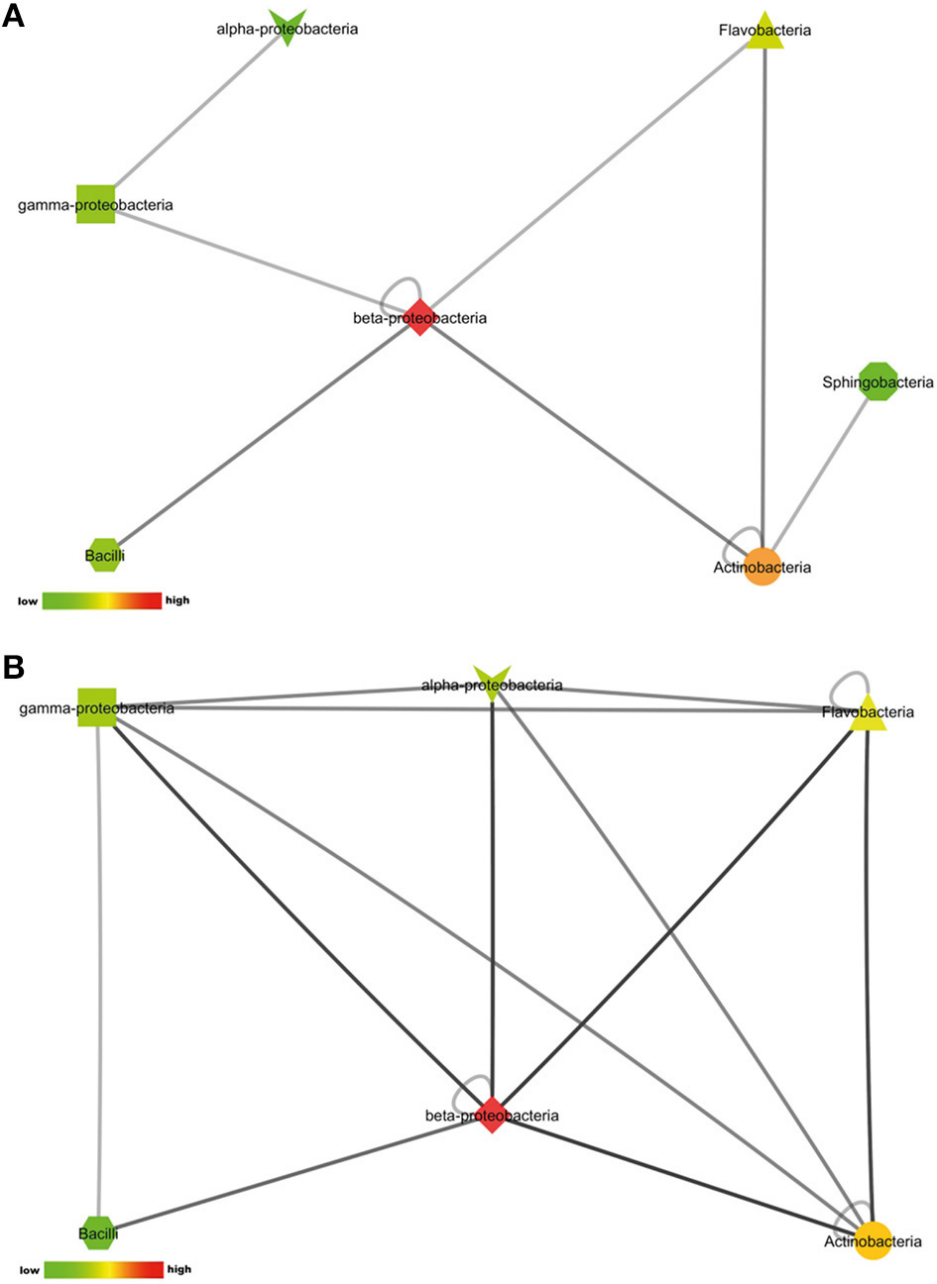
**Interactions between phylogenetic classes that induced antimicrobial activity against (A) the Gram-negative target organism *E. coli* WA321, or (B) against the Gram-positive target organism *S. aureus* 533R4**. Node colors are scaled to the number of interactions between the phylogenetic classes, low number of interactions in bright green, high number of interactions in dark red (see color bar).

The number of pair-wise combinations with induced antimicrobial activity against *S. aureus* 533R4 was higher than against *E. coli* WA321. Most combinations with induced activity against *E. coli* WA321 involved beta-Proteobacteria, Actinobacteria, Flavobacteria, and Bacilli (Figure 4A). Combinations with induced activity against *S. aureus* 533R4 involved all classes of Proteobacteria, Actinobacteria, Flavobacteria, and Bacilli (Figure 4B). Two phylogenetic classes, Flavobacteria and alpha–Proteobacteria, were 3 times more represented in pair-wise combinations with antimicrobial activity than in monocultures (Table 1).

#### Interactions suppressing antimicrobial activity against E. coli or S. aureus

**Figure 5:**
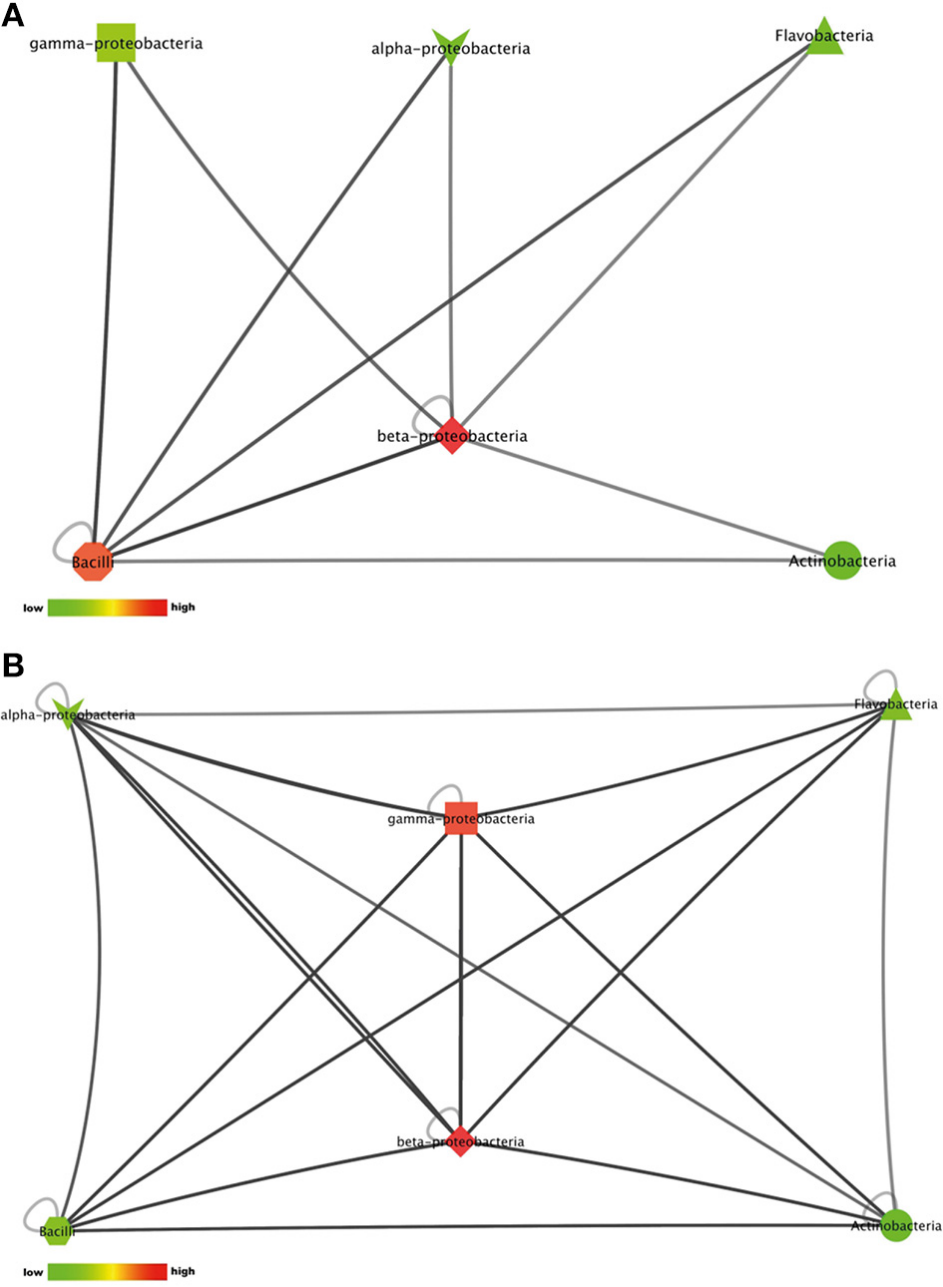
**Interactions between phylogenetic classes that inhibited antimicrobial activity against (A) the Gram-negative target organism *E. coli* WA321, or (B) against the Gram-positive target organism *S. aureus* 533R4**. Node colors are scaled to the respective number of interactions between the phylogenetic classes (low number of interactions in bright colors, high number of interactions in dark colors).

22% of the isolates with antimicrobial activity in monoculture lost this activity during interactions. This apparent suppression of antimicrobial activity was found among all bacterial classes included in this study (Figures 5A,B). Suppression of antimicrobial activity was more frequently found for *S. aureus* than for *E. coli* (Figure 3B). The lists of bacterial pairs which suppressed antimicrobial activity against *S. aureus* and/or *E. coli* are shown in Tables S9, S10.

## Discussion

Recent studies indicated the importance of interspecific bacterial interactions for triggering antibiotic production (Garbeva et al., 2011a; Seyedsayamdost et al., 2012). However, the frequency of such events in natural bacterial communities is not known. Our study focused on a collection of bacterial isolates from similar soil habitats, i.e., sandy soils covered by vegetation patches consisting of sand sedge (*C. arenaria*). Hence, the chance that actual interactions between these bacteria can occur in their natural habitat seems plausible. Induction of antibiotic production in pair-wise combinations was not found to be an abundant phenomenon as it occurred in ~6% of all interactions studied. Yet, 42% of the bacterial isolates were present in combinations that showed activity against at least one of the target organisms, whereas they did not show activity in monocultures. This seems to indicate that the composition of the interacting pairs is an important factor in the induction of antibiotic production.

The observed frequency of interaction-mediated induction of antibiotic production exemplifies that a high-throughput screening as the one developed here can be an important strategy for the discovery of novel cryptic antibiotics. Many pair-wise combinations have to be screened and, subsequently, interesting pairs can be studied in more detail with respect to elucidate the mechanisms underlying the induction, signals and genes involved in the production of the antibiotic compounds (Garbeva et al., 2011a; Traxler et al., 2013). Interactions that induced antimicrobial activity often involved combinations of phylogenetically different bacteria or interactions among beta-Proteobacteria and among Actinobacteria. The present work included several bacterial genera (e.g., *Streptomyces*, *Burkholderia*, *Janthinobacterium* and *Paenibacillus*) for which multiple antibiotics have been described previously (Pantanella et al., 2007; Berdy, 2012; Cornforth and Foster, 2013; Debois et al., 2013; Zhu, 2014). Hence, there is the possibility that our screening method will reveal bacteria that produce known antibiotics but only during co-cultivation.

Few bacterial isolates of the classes Flavobacteria and alpha- Proteobacteria showed antimicrobial activity in monoculture, whereas several strains were present in antibiotic producing combinations. Hence, for these groups there is a clear potential to discover novel antibiotics. Of the 146 tested isolates, 33% showed antimicrobial activity in monoculture. This obtained frequency is in line with previous studies on frequencies of antimicrobial activity in *Streptomyces* spp. (Davelos et al., 2004; Kinkel et al., 2014). However, in many cases antibiotic production was lost when the strain was combined with another strain and only a small percentage (13%) kept their antimicrobial activity in both combinations and monoculture. This suppressing effect on antibiotic production was more often found (22% of all combinations) than the induction of antibiotic production (~6% of all combinations). Several mechanisms can be responsible for the observed suppression of antimicrobial activity during interactions e.g., interference with the quorum sensing system or other signal transduction pathways involved in regulating antibiotic production (Gonzalez and Keshavan, 2006; Venturi and Subramoni, 2009; Christensen et al., 2013) or direct growth inhibition of the antibiotic producing strain (Straight et al., 2007; Hibbing et al., 2010; Schneider et al., 2012). Another possible reason for the observed inhibition of antimicrobial activity during interactions could be lower nutrient availability for each strain during co-cultivation. Growth conditions and nutrient availability are important factors affecting the production of antimicrobial compounds in bacteria (van Wezel and McDowall, 2011). Antibiotic resistance mechanisms might also play a role in the observed inhibition of antimicrobial activity during co-cultivation (Rice, 2006; Wellington et al., 2013).

Depending on the target organism there was a clear difference in antimicrobial activity with higher activity against the Gram-positive than against the Gram-negative organism (in both monocultures and interactions), which is in line with previous reports that Gram-positive bacteria are generally more sensitive to antibiotics (Rice, 2006; Giske et al., 2008; Zhu, 2014).

Soil and rhizosphere are environments where bacteria evolved the ability to produce antibiotics as competitive tool for their survival (Hibbing et al., 2010). Root-associated bacteria with antimicrobial potential play an important role in plant health (Raaijmakers and Mazzola, 2012) and understanding microbial interactions affecting antimicrobial activity may be helpful in understanding the functions and mechanisms of microbial communities contributing to plant protection. The knowledge obtained here could help in selecting the right players in microbial consortia and as suggested by Mendes (Mendes et al., 2013) to design “a minimal microbiome” that comprises a set of microorganisms needed to fulfill a specific ecosystem services like e.g., disease suppression.

In conclusion, the high-throughput screening method developed in this work allows for a fast detection of interaction-mediated induction or suppression of antibiotic production in soil bacteria. Such screening also allows for a better insight into different interference competitive strategies that are operational in microbial communities. This knowledge in turn can be used for construction of synthetic microbial communities (Shong et al., 2012; De Roy et al., 2013; Grosskopf and Soyer, 2014).

### Conflict of interest statement

The authors declare that the research was conducted in the absence of any commercial or financial relationships that could be construed as a potential conflict of interest.

## References

[B1] Altschul S. F. Madden T. L. Schaffer A. A. Zhang J. H. Zhang Z. Miller W.. (1997). Gapped BLAST and PSI-BLAST: a new generation of protein database search programs. Nucleic Acids Res. 25, 3389–3402. 10.1093/nar/25.17.3389 9254694 PMC146917

[B2] Berdy J. (2012). Thoughts and facts about antibiotics: where we are now and where we are heading. J. Antibiot. 65, 385–395. 10.1038/ja.2012.27 22511224

[B3] Chiang Y. M. Chang S. L. Oakley B. R. Wang C. C. C. (2011). Recent advances in awakening silent biosynthetic gene clusters and linking orphan clusters to natural products in microorganisms. Curr. Opin. Chem. Biol. 15, 137–143. 10.1016/j.cbpa.2010.10.011 21111669 PMC3117463

[B4] Christensen L. D. van Gennip M. Rybtke M. T. Wu H. Chiang W. C. Alhede M.. (2013). Clearance of *Pseudomonas aeruginosa* foreign-body biofilm infections through reduction of the cyclic Di-GMP level in bacteria. Infect. Immun. 81, 2705–2731. 10.1128/IAI.00332-13 23690403 PMC3719571

[B5] Cornforth D. M. Foster K. R. (2013). Competition sensing: the social side of bacterial stress responses. Nat. Rev. Microbiol. 11, 285–293. 10.1038/nrmicro2977 23456045

[B6] Dantas G. Sommer M. O. A. Oluwasegun R. D. Church G. M. (2008). Bacteria subsisting on antibiotics. Science 320, 100–103. 10.1126/science.1155157 18388292

[B7] Davelos A. L. Kinkel L. L. Samac D. A. (2004). Spatial variation in frequency and intensity of antibiotic interactions among Streptomycetes from prairie soil. Appl. Environ. Microbiol. 70, 1051–1058. 10.1128/AEM.70.2.1051-1058.2004 14766588 PMC348876

[B8] D'costa V. M. McGrann K. M. Hughes D. W. Wright G. D. (2006). Sampling the antibiotic resistome. Science 311, 374–377. 10.1126/science.1120800 16424339

[B9] De Boer W. Wagenaar A. M. Klein Gunnewiek P. J. A. van Veen J. A. (2007b). *In vitro* suppression of fungi caused by combinations of apparently non-antagonistic soil bacteria. FEMS Microbiol. Ecol. 59, 177–185. 10.1111/j.1574-6941.2006.00197.x 17233750

[B10] De Boer W. Wagenaar A. M. Klein Gunnewiek P. J. van Veen J. A. (2007a). *In vitro* suppression of fungi caused by combinations of apparently non-antagonistic soil bacteria. FEMS Microbiol. Ecol. 59, 177–185. 10.1111/j.1574-6941.2006.00197.x 17233750

[B11] Debois D. Ongena M. Cawoy H. De Pauw E. (2013). MALDI-FTICR MS Imaging as a powerful tool to identify Paenibacillus antibiotics involved in the inhibition of plant pathogens. J. Am. Soc. Mass Spectrom. 24, 1202–1213. 10.1007/s13361-013-0620-2 23636858

[B12] Demoling F. Figueroa D. Baath E. (2007). Comparison of factors limiting bacterial growth in different soils. Soil Biol. Biochem. 39, 2485–2495. 10.1016/j.soilbio.2007.05.002

[B13] De Ridder-Duine A. S. Kowalchuk G. A. Klein Gunnewiek P. J. A. Smant W. van Veen J. A. De Boer W. (2005). Rhizosphere bacterial community composition in natural stands of *Carex arenaria* (sand sedge) is determined by bulk soil community composition. Soil Biol. Biochem. 37, 349–357. 10.1016/j.soilbio.2004.08.005

[B14] De Roy K. Marzorati M. Van den Abbeele P. Van de Wiele T. Boon N. (2013). Synthetic microbial ecosystems: an exciting tool to understand and apply microbial communities. Environ. Microbiol. 16, 1472–1481. 10.1111/1462-2920.12343 24274586

[B15] Edwards U. Rogall T. Blocker H. Emde M. Bottger E. C. (1989). Isolation and direct complete nucleotide determination of entire genes - Characterization of a gene coding for 16s-ribosomal rna. Nucleic Acids Res. 17, 7843–7853. 10.1093/nar/17.19.7843 2798131 PMC334891

[B16] Firn R. D. Jones C. G. (2003). Natural products - a simle model to explain chemical diversity. Nat. Prod. Rep. 20, 382–391. 10.1039/b208815k 12964834

[B17] Gans J. Wolinsky M. Dunbar J. (2005). Computational improvements reveal great bacterial diversity and high metal toxicity in soil. Science 309, 1387–1390. 10.1126/science.1112665 16123304

[B18] Garbeva P. De Boer W. (2009). Inter-specific interactions between carbon-limited soil bacteria affect behavior and gene expression. Microb. Ecol. 58, 36–46. 10.1007/s00248-009-9502-3 19267150

[B19] Garbeva P. Silby M. W. Raaijmakers J. M. Levy S. B. Boer W. (2011a). Transcriptional and antagonistic responses of *Pseudomonas fluorescens* Pf0-1 to phylogenetically different bacterial competitors. ISME J. 5, 973–985. 10.1038/ismej.2010.196 21228890 PMC3131853

[B20] Garbeva P. Tyc O. Remus-Emsermann M. N. P. van der Wal A. Vos M. Silby M.. (2011b). No apparent costs for facultative antibiotic production by the soil bacterium *Pseudomonas fluorescens* Pf0-1. PLoS ONE 6:e27266. 10.1371/journal.pone.0027266 22110622 PMC3217935

[B21] Giske C. G. Monnet D. L. Cars O. Carmeli Y. (2008). Clinical and economic impact of common multidrug-resistant gram-negative bacilli. Antimicrob. Agents Chemother. 52, 813–821. 10.1128/AAC.01169-07 18070961 PMC2258516

[B22] Goh E. B. Yim G. Tsui W. McClure J. Surette M. G. Davies J. (2002). Transcriptional modulation of bacterial gene expression by subinhibitory concentrations of antibiotics. Proc. Natl. Acad. Sci. U.S.A. 99, 17025–17030. 10.1073/pnas.252607699 12482953 PMC139263

[B23] Gonzalez J. E. Keshavan N. D. (2006). Messing with bacterial quorum sensing. Microbiol. Mol. Biol. Rev. 70, 859–875. 10.1128/MMBR.00002-06 17158701 PMC1698510

[B24] Grosskopf T. Soyer O. S. (2014). Synthetic microbial communities. Curr. Opin. Microbiol. 18, 72–77. 10.1016/j.mib.2014.02.002 24632350 PMC4005913

[B25] Hibbing M. E. Fuqua C. Parsek M. R. Peterson S. B. (2010). Bacterial competition: surviving and thriving in the microbial jungle. Nat. Rev. Microbiol. 8, 15–25. 10.1038/nrmicro2259 19946288 PMC2879262

[B26] Ikeda H. Ishikawa J. Hanamoto A. Shinose M. Kikuchi H. Shiba T.. (2003). Complete genome sequence and comparative analysis of the industrial microorganism Streptomyces avermitilis. Nat. Biotechnol. 21, 526–531. 10.1038/nbt820 12692562

[B27] Kinkel L. L. Schlatter D. C. Xiao K. Baines A. D. (2014). Sympatric inhibition and niche differentiation suggest alternative coevolutionary trajectories among Streptomycetes. Isme J. 8, 249–256. 10.1038/ismej.2013.175 24152720 PMC3906824

[B28] Lane D. J. (1991). 16S/23S rRNA sequencing, in Nucleic Acid Techniques in Bacterial Systematics, eds Stackebrandt E. Goodfellow M. (New York, NY: John Wiley and Sons), 15–175

[B29] Linares J. F. Gustafsson I. Baquero F. Martinez J. L. (2006). Antibiotics as intermicrobial signaling agents instead of weapons. Proc. Natl. Acad. Sci. U.S.A. 103, 19484–19489. 10.1073/pnas.0608949103 17148599 PMC1682013

[B30] Lyon G. J. Muir T. W. (2003). Chemical signaling among bacteria and its inhibition. Chem. Biol. 10, 1007–1021. 10.1016/j.chembiol.2003.11.003 14652068

[B31] Maurhofer M. Baehler E. Notz R. Martinez V. Keel C. (2004). Cross talk between 2,4-diacetylphloroglucinol-producing biocontrol pseudomonads on wheat roots. Appl. Environ. Microbiol. 70, 1990–1998. 10.1128/AEM.70.4.1990-1998.2004 15066789 PMC383149

[B32] Mendes R. Garbeva P. Raaijmakers J. M. (2013). The rhizosphere microbiome: significance of plant beneficial, plant pathogenic, and human pathogenic microorganisms. FEMS Microbiol. Rev. 37, 634–663. 10.1111/1574-6976.12028 23790204

[B33] Nkanga E. J. Hagedorn C. (1978). Detection of antibiotic-producing Streptomyces inhabiting forest soils. Antimicrob. Agents Chemother. 14, 51–59. 10.1128/AAC.14.1.51 686709 PMC352404

[B34] Pantanella F. Berlutti F. Passariello C. Sarli S. Morea C. Schippa S. (2007). Violacein and biofilm production in Janthinobacterium lividum. J. Appl. Microbiol. 102, 992–999. 10.1111/j.1365-2672.2006.03155.x 17381742

[B35] Raaijmakers J. M. Mazzola M. (2012). Diversity and natural functions of antibiotics produced by beneficial and plant pathogenic bacteria. Annu. Rev. Phytopathol. 50, 403–424. 10.1146/annurev-phyto-081211-172908 22681451

[B36] Rice L. B. (2006). Antimicrobial resistance in gram-positive bacteria. Am. J. Infect. Control 34, S11–S19, discussion; S64–S73. 10.1016/j.ajic.2006.05.220 16813977

[B37] Romero D. Traxler M. F. Lopez D. Kolter R. (2011). Antibiotics as signal molecules. Chem. Rev. 111, 5492–5505. 10.1021/cr2000509 21786783 PMC3173521

[B38] Rousk J. Baath E. (2007). Fungal and bacterial growth in soil with plant materials of different C/N ratios. FEMS Microbiol. Ecol. 62, 258–267. 10.1111/j.1574-6941.2007.00398.x 17991019

[B39] Rousk J. Demoling L. A. Baath E. (2009). Contrasting short-term antibiotic effects on respiration and bacterial growth compromises the validity of the selective respiratory inhibition technique to distinguish fungi and bacteria. Microb. Ecol. 58, 75–85. 10.1007/s00248-008-9444-1 18797957

[B40] Saleh O. Bonitz T. Flinspach K. Kulik A. Burkard N. Muhlenweg A.. (2012). Activation of a silent phenazine biosynthetic gene cluster reveals a novel natural product and a new resistance mechanism against phenazines. Medchemcomm 3, 1009–1019. 10.1039/c2md20045g

[B41] Sambrook J. Russell D. W. (2001). Molecular Cloning: A Laboratory Manual. 3rd Edn. Plainview, NY: Cold Spring Harbor Laboratory Press

[B42] Scherlach K. Hertweck C. (2009). Triggering cryptic natural product biosynthesis in microorganisms. Org. Biomol. Chem. 7, 1753–1760. 10.1039/b821578b 19590766

[B43] Schneider J. Yepes A. Garcia-Betancur J. C. Westedt I. Mielich B. Lopez D. (2012). Streptomycin-induced expression in *Bacillus subtilis* of YtnP, a lactonase-homologous protein that inhibits development and streptomycin production in *Streptomyces griseus*. Appl. Environ. Microbiol. 78, 599–603. 10.1128/AEM.06992-11 22101040 PMC3255736

[B44] Seyedsayamdost M. R. Traxler M. F. Clardy J. Kolter R. (2012). Old meets new: using interspecies interactions to detect secondary metabolite production in actinomycetes. Methods Enzymol. 517, 89–109. 10.1016/B978-0-12-404634-4.00005-X 23084935 PMC4004031

[B45] Shannon P. Markiel A. Ozier O. Baliga N. S. Wang J. T. Ramage D.. (2003). Cytoscape: a software environment for integrated models of biomolecular interaction networks. Genome Res. 13, 2498–2504. 10.1101/gr.1239303 14597658 PMC403769

[B46] Shong J. Jimenez Diaz M. R. Collins C. H. (2012). Towards synthetic microbial consortia for bioprocessing. Curr. Opin. Biotechnol. 23, 798–802. 10.1016/j.copbio.2012.02.001 22387100

[B47] Slattery M. Rajbhandari I. Wesson K. (2001). Competition-mediated antibiotic induction in the marine bacterium *Streptomyces tenjimariensis*. Microb. Ecol. 41, 90–96. 10.1007/s002480000084 12032613

[B48] Straight P. D. Fischbach M. A. Walsh C. T. Rudner D. Z. Kolter R. (2007). A singular enzymatic megacomplex from *Bacillus subtilis*. Proc. Natl. Acad. Sci. U.S.A. 104, 305–310. 10.1073/pnas.0609073103 17190806 PMC1765455

[B49] Strickland M. S. Lauber C. Fierer N. Bradford M. A. (2009). Testing the functional significance of microbial community composition. Ecology 90, 441–451. 10.1890/08-0296.1 19323228

[B50] Traxler M. F. Watrous J. D. Alexandrov T. Dorrestein P. C. Kolter R. (2013). Interspecies interactions stimulate diversification of the *Streptomyces coelicolor* secreted metabolome. MBio 4:e00459-13. 10.1128/mBio.00459-13 23963177 PMC3747584

[B51] Uroz S. Buee M. Murat C. Frey-Klett P. Martin F. (2010). Pyrosequencing reveals a contrasted bacterial diversity between oak rhizosphere and surrounding soil. Environ. Microbiol. Rep. 2, 281–288. 10.1111/j.1758-2229.2009.00117.x 23766079

[B52] van Wezel G. P. McDowall K. J. (2011). The regulation of the secondary metabolism of Streptomyces: new links and experimental advances. Nat. Prod. Rep. 28, 1311–1333. 10.1039/c1np00003a 21611665

[B53] Venturi V. Subramoni S. (2009). Future research trends in the major chemical language of bacteria. HFSP J. 3, 105–116. 10.2976/1.3065673 19794815 PMC2707791

[B54] Wellington E. M. Boxall A. B. Cross P. Feil E. J. Gaze W. H. Hawkey P. M.. (2013). The role of the natural environment in the emergence of antibiotic resistance in gram-negative bacteria. Lancet Infect. Dis. 13, 155–165. 10.1016/S1473-3099(12)70317-1 23347633

[B55] Yin B. Crowley D. Sparovek G. De Melo W. J. Borneman J. (2000). Bacterial functional redundancy along a soil reclamation gradient. Appl. Environ. Microbiol. 66, 4361–4365. 10.1128/AEM.66.10.4361-4365.2000 11010883 PMC92309

[B56] Zhu H. (2014). Environmental and Metabolomic Study of Antibiotic Production by Actinomycetes. Ph.D. Thesis, Leiden University, The Netherlands

