# Supplementary material for: Impact of interspecific interactions on antimicrobial activity among soil bacteria
Source: Front Microbiol. 2014 Oct 28;5:567. doi: 10.3389/fmicb.2014.00567 (PMC4211544; doi:10.3389/fmicb.2014.00567)
Supplement: Supplementary file 1 [file DataSheet1.PDF]

Supplementary Material

Table S1 Results of antimicrobial screening in confrontation assays for all bacterial isolates used in this study.

Phylum / phylogenetic class	Species	Accession number	isolation medium	AM activity vs *E.coli*		AM activity vs *S.aureus*	
**Actinobacteria**				Monoculture	Interaction	Monoculture	Interaction
Actinobacteria	*Micrococcus* sp. AD12	KJ685229	TSBA	x		x	
Actinobacteria	*Rhodococcus* sp. AD22	KJ685237	TSBA			x	x
Actinobacteria	*Streptomyces* sp. AD29	KJ685244	TSBA	x		x	
Actinobacteria	*Micrococcus* sp. AD31	KJ685246	TSBA		x (1)		x (9)
Actinobacteria	*Streptomyces* sp. AD92	KJ685303	CHIT				x (4)
Actinobacteria	*Streptomyces* sp. AD94	KJ685305	CHIT			x	x
Actinobacteria	*Streptomyces* sp. AD107	KJ685318	TSBA			x	
Actinobacteria	*Streptomyces* sp. AD108	KJ685319	TSBA		x (4)		x (8)
Actinobacteria	*Mycobacterium* sp. AD110	KJ685321	TSBA	x		x	
Actinobacteria	*Microbacterium* sp. AD141	KJ685346	TSBA		x (2)		x (3)
Actinobacteria	*Tsukamurella* sp. AD106	KJ685317	TSBA				x (3)
**Bacteroidetes**							
Flavobacteria	*Flavobacterium* sp. AD41	KJ685256	TSBA			x	
Flavobacteria	*Flavobacterium* sp. AD42	KJ685257	TSBA				x (1)
Flavobacteria	*Flavobacterium* sp. AD43	KJ685258	TSBA				x (1)
Flavobacteria	*Flavobacterium* sp. AD44	KJ685259	TSBA				x (1)
Flavobacteria	*Flavobacterium* sp. AD45	KJ685260	TSBA				x (1)
Flavobacteria	*Flavobacterium* sp. AD47	KJ685262	TSBA		x (1)	x	x
Flavobacteria	*Chryseobacterium* sp. AD48	KJ685263	TSBA				x (3)
Flavobacteria	*Flavobacterium* sp. AD84	KJ685296	CHIT		x (1)	x	
Flavobacteria	*Flavobacterium* sp. AD85	KJ685297	CHIT				x (1)
Flavobacteria	*Flavobacterium* sp. AD86	KJ685298	CHIT	x		x	
Flavobacteria	*Flavobacterium* sp. AD91	KJ685302	CHIT				x (2)
Flavobacteria	*Flavobacterium* sp. AD131	KJ685338	TSBA				
Flavobacteria	*Flavobacterium* sp. AD134	KJ685341	TSBA				
Flavobacteria	*Flavobacterium* sp. AD142	KJ685347	TSBA				x (1)
Flavobacteria	*Flavobacterium* sp. AD146	KJ685351	CHIT				x (2)
Flavobacteria	*Flavobacterium* sp. AD149	KJ685354	TSBA				
Flavobacteria	*Flavobacterium* sp. AD151	KJ685355	TSBA		x (1)		
Flavobacteria	*Flavobacterium* sp. AD155	KJ685358	CHIT				x (1)
Flavobacteria	*Flavobacterium* sp. AD156	KJ685359	CHIT				x (1)
Sphingobacteria	*Pedobacter* sp. V48	DQ778037	TSBA		x (1)		
**Firmicutes**							
Bacilli	*Paenibacillus* sp. AD50	KJ685264	TSBA	x			
Bacilli	*Bacillus* sp. AD78	KJ685290	CHIT		x (1)	x	
Bacilli	*Paenibacillus* sp. AD83	KJ685295	CHIT		x (1)		x (2)
Bacilli	*Paenibacillus* sp. AD87	KJ685299	CHIT			x	
Bacilli	*Paenibacillus* sp. AD93	KJ685304	CHIT			x	
Bacilli	*Paenibacillus* sp. AD116	KJ685325	TSBA				x (1)
Bacilli	*Paenibacillus* sp. AD117	KJ685326	TSBA	x			
**Proteobacteria**							
alpha-proteobacteria	*Agrobacterium* sp. AD1	KJ685218	TSBA				
alpha-proteobacteria	*Phyllobacterium* sp. AD34	KJ685249	TSBA				x (1)
alpha-proteobacteria	*Mesorhizobium* sp. AD38	KJ685253	TSBA				x (1)
alpha-proteobacteria	*Mesorhizobium* sp. AD112	KJ685322	TSBA				x (1)
alpha-proteobacteria	*Bosea* sp. AD113	KJ685323	TSBA			x	
alpha-proteobacteria	*Bradyrhizobiaceae* sp. AD126	KJ685334	TSBA				
alpha-proteobacteria	*Bosea* sp. AD132	KJ685339	TSBA				x (1)
alpha-proteobacteria	*Agrobacterium* sp. AD140	KJ685345	TSBA				x (1)
alpha-proteobacteria	*Rhizobium* sp. AD148	KJ685353	TSBA				x (1)
alpha-proteobacteria	*Phyllobacterium* sp. AD136	KJ685342	TSBA				x (2)
alpha-proteobacteria	*Phyllobacterium* sp. AD152	KJ685356	TSBA		x (1)	x	
alpha-proteobacteria	*Phyllobacterium* sp. AD153	KJ685357	TSBA				x (4)
alpha-proteobacteria	*Phyllobacterium* sp. AD159	KJ685361	TSBA				x (1)
alpha-proteobacteria	*Phyllobacterium* sp. AD51	KJ685265	CHIT			x	
beta-proteobacteria	*Burkholderia* sp. AD3	KJ685220	TSBA				
beta-proteobacteria	*Burkholderia* sp. AD9	KJ685226	TSBA			x	
beta-proteobacteria	*Burkholderia* sp. AD10	KJ685227	TSBA	x		x	
beta-proteobacteria	*Burkholderia* sp. AD15	KJ685231	TSBA	x		x	x
beta-proteobacteria	*Burkholderia* sp. AD11	KJ685228	TSBA		x (2)	x	
beta-proteobacteria	*Burkholderia* sp. AD18	KJ685234	TSBA				x (2)
beta-proteobacteria	*Collimonas* sp. AD19	KJ685235	TSBA			x	
beta-proteobacteria	*Collimonas* sp. AD23	KJ685238	TSBA	x		x	
beta-proteobacteria	*Burkholderia* sp. AD24	KJ685239	TSBA		x (2)		x (1)
beta-proteobacteria	*Burkholderia* sp. AD25	KJ685240	TSBA	x		x	x
beta-proteobacteria	*Burkholderia* sp. AD26	KJ685241	TSBA	x		x	
beta-proteobacteria	*Burkholderia* sp. AD27	KJ685242	TSBA	x		x	x
beta-proteobacteria	*Burkholderia* sp. AD28	KJ685243	TSBA	x		x	
beta-proteobacteria	*Burkholderia* sp. AD30	KJ685245	TSBA	x		x	
beta-proteobacteria	*Burkholderia* sp. AD32	KJ685247	TSBA		x (1)	x	
beta-proteobacteria	*Collimonas* sp. AD33	KJ685248	TSBA	x		x	
beta-proteobacteria	*Burkholderia* sp. AD35	KJ685250	TSBA			x	
beta-proteobacteria	*Burkholderia* sp. AD37	KJ685252	TSBA		x (1)		x (5)
beta-proteobacteria	*Variovorax* sp. AD39	KJ685254	TSBA			x	

Table S1 continuation

Phylum / phylogenetic class	Species	Accession number	isolation medium	AM activity vs *E.coli*		AM activity vs *S.aureus*	
**Proteobacteria**				Monoculture	Interaction	Monoculture	Interaction
beta-proteobacteria	*Janthinobacterium* sp. AD54	KJ685267	TSBA			x	
beta-proteobacteria	*Janthinobacterium* sp. AD55	KJ685268	TSBA	x			
beta-proteobacteria	*Collimonas* sp. AD58	KJ685270	CHIT				
beta-proteobacteria	*Collimonas* sp. AD59	KJ685271	CHIT				
beta-proteobacteria	*Collimonas* sp. AD60	KJ685272	CHIT				
beta-proteobacteria	*Collimonas* sp. AD61	KJ685273	CHIT				x (1)
beta-proteobacteria	*Collimonas* sp. AD62	KJ685274	CHIT				x (3)
beta-proteobacteria	*Collimonas* sp. AD63	KJ685275	CHIT			x	x
beta-proteobacteria	*Collimonas* sp. AD64	KJ685276	CHIT			x	
beta-proteobacteria	*Collimonas* sp. AD65	KJ685277	CHIT				x (5)
beta-proteobacteria	*Collimonas* sp. AD66	KJ685278	CHIT			x	
beta-proteobacteria	*Collimonas* sp. AD67	KJ685279	CHIT				x (2)
beta-proteobacteria	*Collimonas* sp. AD68	KJ685280	CHIT		x (1)		x (1)
beta-proteobacteria	*Collimonas* sp. AD69	KJ685281	CHIT				x (3)
beta-proteobacteria	*Collimonas* sp. AD70	KJ685282	CHIT				x (1)
beta-proteobacteria	*Collimonas* sp. AD71	KJ685283	CHIT				x (1)
beta-proteobacteria	*Janthinobacterium* sp. AD72	KJ685284	CHIT		x (1)		x (3)
beta-proteobacteria	*Janthinobacterium* sp. AD73	KJ685285	CHIT				x (1)
beta-proteobacteria	*Janthinobacterium* sp. AD74	KJ685286	CHIT	x		x	
beta-proteobacteria	*Janthinobacterium* sp. AD75	KJ685287	CHIT				x (2)
beta-proteobacteria	*Collimonas* sp. AD76	KJ685288	CHIT			x	x
beta-proteobacteria	*Collimonas* sp. AD77	KJ685289	CHIT	x		x	
beta-proteobacteria	*Janthinobacterium* sp. AD80	KJ685292	CHIT		x (4)	x	
beta-proteobacteria	*Silvimonas* sp. AD81	KJ685293	CHIT			x	
beta-proteobacteria	*Silvimonas* sp. AD82	KJ685294	CHIT			x	
beta-proteobacteria	*Collimonas* sp. AD88	KJ685300	CHIT				x (3)
beta-proteobacteria	*Collimonas* sp. AD89	KJ685301	CHIT				x (3)
beta-proteobacteria	*Collimonas* sp. AD95	KJ685306	CHIT	x		x	
beta-proteobacteria	*Janthinobacterium* sp. AD96	KJ685307	CHIT	x		x	
beta-proteobacteria	*Collimonas* sp. AD97	KJ685308	CHIT				x (2)
beta-proteobacteria	*Collimonas* sp. AD98	KJ685309	CHIT				x (5)
beta-proteobacteria	*Collimonas* sp. AD99	KJ685310	CHIT				x (1)
beta-proteobacteria	*Collimonas* sp. AD101	KJ685312	CHIT				x (1)
beta-proteobacteria	*Collimonas* sp. AD102	KJ685313	CHIT				x (1)
beta-proteobacteria	*Collimonas* sp. AD103	KJ685314	CHIT			x	
beta-proteobacteria	*Janthinobacterium* sp. AD118	KJ685327	TSBA	x		x	x
beta-proteobacteria	*Janthinobacterium* sp. AD119	KJ685328	TSBA	x		x	
beta-proteobacteria	*Burkholderia* sp. AD123	KJ685331	TSBA				
beta-proteobacteria	*Burkholderia* sp. AD127	KJ685335	TSBA	x			
beta-proteobacteria	*Variovorax* sp. AD130	KJ685337	TSBA				
beta-proteobacteria	*Variovorax* sp. AD133	KJ685340	TSBA		x (1)	x	
beta-proteobacteria	*Collimonas* sp. AD137	KJ685343	TSBA				x (1)
beta-proteobacteria	*Burkholderia* sp. AD138	KJ685344	TSBA				x (1)
beta-proteobacteria	*Variovorax* sp. AD143	KJ685348	TSBA				x (5)
beta-proteobacteria	*Janthinobacterium* sp. AD144	KJ685349	CHIT			x	
beta-proteobacteria	*Roseateles* sp. AD145	KJ685350	CHIT				x (1)
gamma-proteobacteria	*Pseudomonas* sp. AD2	KJ685219	TSBA				
gamma-proteobacteria	*Pseudomonas* sp. AD4	KJ685221	TSBA			x	
gamma-proteobacteria	*Pseudomonas* sp. AD5	KJ685222	TSBA			x	
gamma-proteobacteria	*Pseudomonas* sp. AD6	KJ685223	TSBA			x	
gamma-proteobacteria	*Pseudomonas* sp. AD7	KJ685224	TSBA			x	x
gamma-proteobacteria	*Pseudomonas* sp. AD8	KJ685225	TSBA			x	
gamma-proteobacteria	*Pseudomonas* sp. AD14	KJ685230	TSBA			x	
gamma-proteobacteria	*Pseudomonas* sp. AD16	KJ685232	TSBA			x	
gamma-proteobacteria	*Pseudomonas* sp. AD17	KJ685233	TSBA			x	
gamma-proteobacteria	*Pseudomonas* sp. AD21	DQ778036	TSBA	x		x	x
gamma-proteobacteria	*Pseudomonas* sp. AD36	KJ685251	TSBA				
gamma-proteobacteria	*Pseudomonas* sp. AD79	KJ685291	CHIT			x	
gamma-proteobacteria	*Pseudomonas* sp. AD100	KJ685311	CHIT			x	x
gamma-proteobacteria	*Pseudomonas* sp. AD104	KJ685315	CHIT				x (5)
gamma-proteobacteria	*Pseudomonas* sp. AD105	KJ685316	CHIT				x (2)
gamma-proteobacteria	*Pseudomonas* sp. AD114	KJ685324	TSBA		x (1)		x (1)
gamma-proteobacteria	*Pseudomonas* sp. AD122	KJ685330	TSBA				
gamma-proteobacteria	*Pseudomonas* sp. AD124	KJ685332	TSBA				x (1)
gamma-proteobacteria	*Pseudomonas* sp. AD125	KJ685333	TSBA	x			
gamma-proteobacteria	*Pseudomonas* sp. AD157	KJ685360	TSBA				x (1)
gamma-proteobacteria	*Luteibactor* sp. AD20	KJ685236	TSBA			x	
gamma-proteobacteria	*Dyella* sp. AD40	KJ685255	TSBA			x	
gamma-proteobacteria	*Dyella* sp. AD46	KJ685261	TSBA				x (1)
gamma-proteobacteria	*Lysobacter* sp. AD52	KJ685266	CHIT			x	
gamma-proteobacteria	*Dyella* sp. AD56	KJ685269	TSBA		x (1)	x	
gamma-proteobacteria	*Frateuria* sp. AD120	KJ685329	TSBA				
gamma-proteobacteria	*Dyella* sp. AD129	KJ685336	TSBA				
gamma-proteobacteria	*Stenotrophomonas* sp. AD147	KJ685352	CHIT				x (1)
gamma-proteobacteria	*Rhodonobacter* sp. AD109	KJ685320	TSBA				

AM activity = antimicrobial activity against *E.coli* WA321 or *S.aureus* 533R4 in monoculture or in interactions. Isolation media: CHIT= chitin supplemented agar plates, TSBA= Tryptic soy broth agar plates. Numbers in brackets represent the number of interactions that triggered antimicrobial activity either against *E.coli* WA321 or *S.aureus* 533R4.

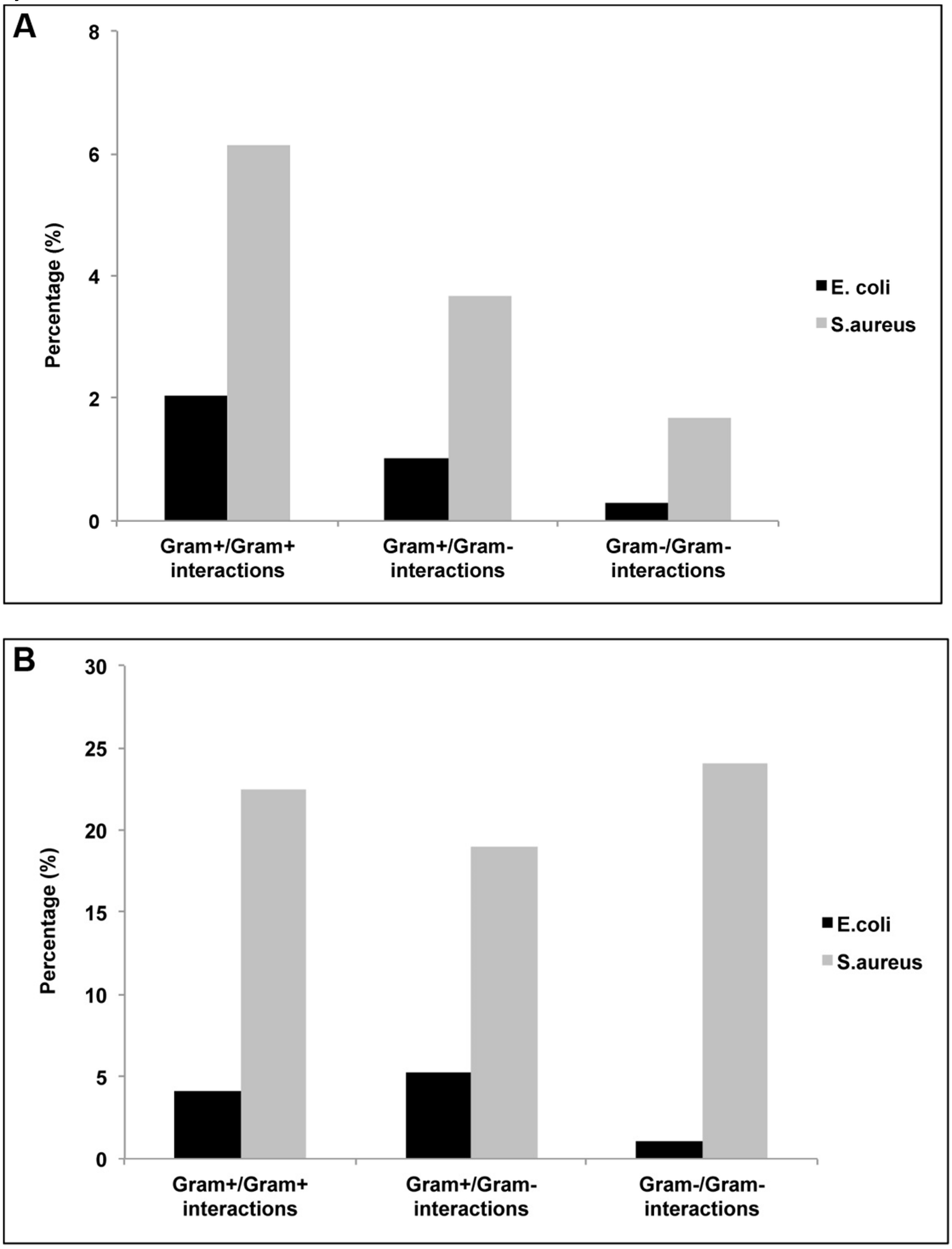

Supplementary Figure S2 Frequencies of induction **(A)** or suppression of **(B)** antimicrobial activities for combinations of Gram- or Gram+ bacterial isolates. Antimicrobial activities were tested against *E.coli* WA321 or *S.aureus* 533R4. Number of tested interactions: Gram+/Gram+: n=49, Gram+/Gram-: n=690, Gram-/Gram-: n=2069. The results of the statistical analyses (Chi-square tests) are presented in **Table S5** and **S6**.

Supplementary Table S3 Bacterial strains used as target and positive control in the high-throughput interaction assay and the agar-overlay assay to detect antimicrobial activity.

Organism	Strain number	Assay function
Streptomyces rimosus	DSMZ 40 260 (type strain)	positive control, produces oxytetracycline
Streptomyces nodosus	DSMZ 40 109 (type strain)	positive control, produces amphotericin A and amphotericin B
Streptomyces kanamyceticus	DSMZ 40 500 (type strain)	positive control, produces kanamycin A,B and C
Escherichia coli WA321	DSMZ 4509	target, model for human pathogen
Staphylococcus aureus 533R4 Serovar 3	DSMZ 20231	target, model for human pathogen

Supplementary Table S4 Bacterial isolates without any antimicrobial activity against *E.coli* WA 321 or *S.aureus* 533R4 either in paired combination or monoculture.

Phylum / phylogenetic class	Species	Accession number	isolation medium	AM activity vs *E.coli*		AM activity vs *S.aureus*	
				Monoculture	Interaction	Monoculture	Interaction
Bacteroidetes							
Flavobacteria	*Flavobacterium* sp. AD131	KJ685338	TSBA	-	-	-	-
Flavobacteria	*Flavobacterium* sp. AD134	KJ685341	TSBA	-	-	-	-
Flavobacteria	*Flavobacterium* sp. AD149	KJ685354	TSBA	-	-	-	-
Proteobacteria							
alpha-proteobacteria	*Agrobacterium* sp. AD1	KJ685218	TSBA	-	-	-	-
alpha-proteobacteria	*Bradyrhizobiaceae* sp. AD126	KJ685334	TSBA	-	-	-	-
beta-proteobacteria	*Burkholderia* sp. AD3	KJ685220	TSBA	-	-	-	-
beta-proteobacteria	*Collimonas* sp. AD58	KJ685270	CHIT	-	-	-	-
beta-proteobacteria	*Collimonas* sp. AD59	KJ685271	CHIT	-	-	-	-
beta-proteobacteria	*Collimonas* sp. AD60	KJ685272	CHIT	-	-	-	-
beta-proteobacteria	*Burkholderia* sp. AD123	KJ685331	TSBA	-	-	-	-
beta-proteobacteria	*Variovorax* sp. AD130	KJ685337	TSBA	-	-	-	-
gamma-proteobacteria	*Pseudomonas* sp. AD2	KJ685219	TSBA	-	-	-	-
gamma-proteobacteria	*Pseudomonas* sp. AD36	KJ685251	TSBA	-	-	-	-
gamma-proteobacteria	*Rhodonobacter* sp. AD109	KJ685320	TSBA	-	-	-	-
gamma-proteobacteria	*Frateuria* sp. AD120	KJ685329	TSBA	-	-	-	-
gamma-proteobacteria	*Pseudomonas* sp. AD122	KJ685330	TSBA	-	-	-	-
gamma-proteobacteria	*Dyella* sp. AD129	KJ685336	TSBA	-	-	-	-

Supplementary Table S5: Results of the Chi-square test for **f**requencies of induction of antimicrobial activity against **(A)** *E.coli* WA321 and **(B)** *S.aureus* 533R4. Abbreviations: Rows: Gram-group interactions (1: Gram-/Gram- interactions, 2: Gram+/Gram- interactions, 3: Gram+/Gram+ interactions). Columns: Number of interactions with A: triggering of antimicrobial activity and B: Interactions without triggering.

A 1.6.2014 Contingency Table: Results

$r \times c$ Contingency Table: Results

The results of a contingency table X^2 statistical test performed at 11:29 on 1-JUN-2014

data: contingency table

	A	B	
1	6	2063	2069
2	7	673	680
3	1	48	49
	14	2784	2798

expected: contingency table

	A	B
1	10.4	2.059E+03
2	3.40	677.
3	0.245	48.8

chi-square = 8.00
degrees of freedom = 2
probability = 0.018

B 1.6.2014 Contingency Table: Results

$r \times c$ Contingency Table: Results

The results of a contingency table X^2 statistical test performed at 11:30 on 1-JUN-2014

data: contingency table

	A	B	
1	35	2034	2069
2	25	655	680
3	3	46	49
	63	2735	2798

expected: contingency table

	A	B
1	46.6	2.022E+03
2	15.3	665.
3	1.10	47.9

chi-square = 12.6
degrees of freedom = 2
probability = 0.002

Supplementary Table S6: Results of the Chi-square test for the frequencies of silencing of antimicrobial activity against **(A)** *E.coli* WA 321 and **(B)** *S.aureus* 533R4. Abbreviations: Rows: Gram-group interactions (1: Gram-/Gram- interactions, 2: Gram+/Gram- interactions, 3: Gram+/Gram+ interactions). Columns: Number of interactions with A: observed silencing of antimicrobial activity and B: without observed silencing.

A 1.6.2014 Contingency Table: Results

$r \times c$ Contingency Table: Results

The results of a contingency table X^2 statistical test performed at 11:33 on 1-JUN-2014

data: contingency table

	A	B	
1	22	2047	2069
2	36	644	680
3	2	47	49
	60	2738	2798

expected: contingency table

	A	B
1	44.4	2.025E+03
2	14.6	665.
3	1.05	47.9

chi-square = 44.5
degrees of freedom = 2
probability = 0.000

B 1.6.2014 Contingency Table: Results

$r \times c$ Contingency Table: Results

The results of a contingency table X^2 statistical test performed at 11:35 on 1-JUN-2014

data: contingency table

	A	B	
1	499	1570	2069
2	129	551	680
3	11	38	49
	639	2159	2798

expected: contingency table

	A	B
1	473.	1.596E+03
2	155.	525.
3	11.2	37.8

chi-square = 7.70
degrees of freedom = 2
probability = 0.021

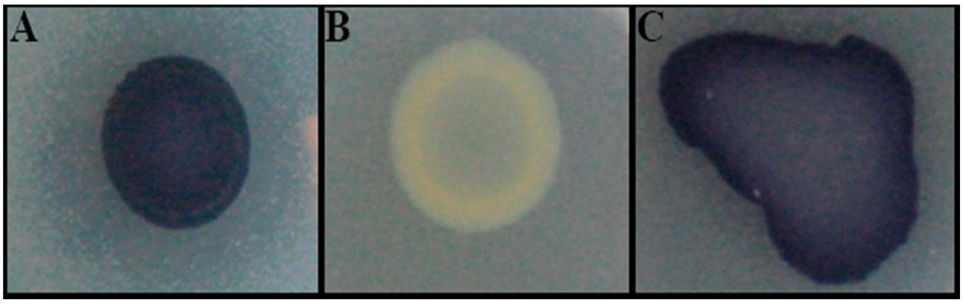

Supplementary Figure S7 Suppression of antimicrobial activity revealed via the agar overlay assay: *Janthinobacterium* sp. AD55 monoculture (**A**), *Flavobacterium* sp. AD86 monoculture (**B**), Interaction *Janthinobacterium* sp. AD55 with *Flavobacterium* sp. AD86 loss of antimicrobial activity against E. *coli* WA321.

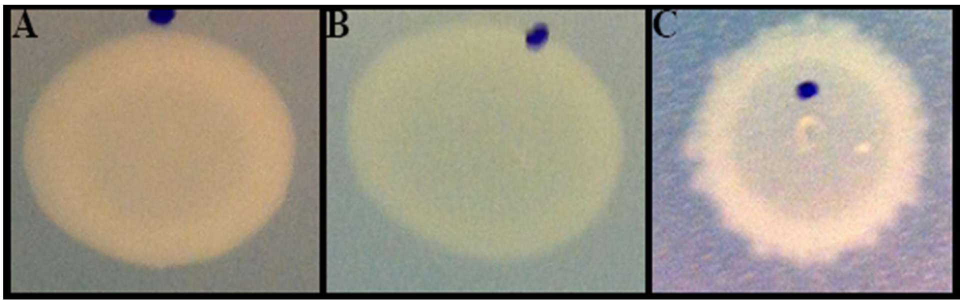

Supplementary Figure S8 Induction of antimicrobial activity revealed via the agar overlay assay: *Dyella* sp. AD56 monoculture (**A**), *Janthinobacterium* sp. AD80 monoculture (**B**), Interaction *Dyella* sp. AD56 with *Janthinobacterium* sp. AD80 antimicrobial activity against E. *coli* WA321.

Supplementary Table S9 Bacterial combination pairs which suppressed antimicrobial activity against *E.coli* WA 321. For the strain designation please see Table S1.

Phylogenetic class	Strain code genus A	Phylogenetic class	Strain code genus B
Bacilli	AD50	beta-proteobacteria	AD10
Bacilli	AD50	Actinobacteria	AD22
Bacilli	AD50	alpha-proteobacteria	AD34
Bacilli	AD50	beta-proteobacteria	AD60
Bacilli	AD50	beta-proteobacteria	AD70
Bacilli	AD50	beta-proteobacteria	AD9
Bacilli	AD50	beta-proteobacteria	AD26
Bacilli	AD50	beta-proteobacteria	AD81
Bacilli	AD50	beta-proteobacteria	AD98
Bacilli	AD50	beta-proteobacteria	AD89
Bacilli	AD50	Bacilli	AD87
Bacilli	AD50	beta-proteobacteria	AD137
Bacilli	AD50	alpha-proteobacteria	AD148
Bacilli	AD50	gamma-proteobacteria	AD157
Bacilli	AD50	Flavobacteria	AD155
Bacilli	AD50	beta-proteobacteria	AD25
Bacilli	AD50	beta-proteobacteria	AD33
Bacilli	AD50	Bacilli	AD83
Bacilli	AD50	beta-proteobacteria	AD101
Bacilli	AD50	gamma-proteobacteria	AD125
Bacilli	AD50	Flavobacteria	AD142
Bacilli	AD117	gamma-proteobacteria	AD20
Bacilli	AD117	Flavobacteria	AD42
Bacilli	AD117	beta-proteobacteria	AD68
Bacilli	AD117	beta-proteobacteria	AD24
Bacilli	AD117	beta-proteobacteria	AD95
Bacilli	AD117	Flavobacteria	AD146
Bacilli	AD117	alpha-proteobacteria	AD159
Bacilli	AD117	gamma-proteobacteria	AD14
Bacilli	AD117	gamma-proteobacteria	AD56
Bacilli	AD117	beta-proteobacteria	AD69
Bacilli	AD117	beta-proteobacteria	AD98
Bacilli	AD117	alpha-proteobacteria	AD001
Bacilli	AD117	Actinobacteria	AD12
Bacilli	AD117	gamma-proteobacteria	AD2
beta-proteobacteria	AD55	beta-proteobacteria	AD11
beta-proteobacteria	AD55	beta-proteobacteria	AD23
beta-proteobacteria	AD55	beta-proteobacteria	AD35
beta-proteobacteria	AD55	Flavobacteria	AD45
beta-proteobacteria	AD55	beta-proteobacteria	AD61
beta-proteobacteria	AD55	beta-proteobacteria	AD71
beta-proteobacteria	AD55	beta-proteobacteria	AD15
beta-proteobacteria	AD55	Actinobacteria	AD22
beta-proteobacteria	AD55	beta-proteobacteria	AD82
beta-proteobacteria	AD55	gamma-proteobacteria	AD100
beta-proteobacteria	AD55	beta-proteobacteria	AD98
beta-proteobacteria	AD55	beta-proteobacteria	AD138
beta-proteobacteria	AD55	beta-proteobacteria	AD143
beta-proteobacteria	AD55	gamma-proteobacteria	AD147
beta-proteobacteria	AD55	beta-proteobacteria	AD10
beta-proteobacteria	AD55	beta-proteobacteria	AD60
beta-proteobacteria	AD55	Flavobacteria	AD86
beta-proteobacteria	AD55	beta-proteobacteria	AD102
beta-proteobacteria	AD55	beta-proteobacteria	AD74
beta-proteobacteria	AD55	alpha-proteobacteria	AD112
beta-proteobacteria	AD55	alpha-proteobacteria	AD126
gamma-proteobacteria	AD125	beta-proteobacteria	AD66
gamma-proteobacteria	AD125	Bacilli	AD50
beta-proteobacteria	AD127	beta-proteobacteria	AD75
beta-proteobacteria	AD127	Actinobacteria	AD107

Supplementary Table S10 Bacterial combination pairs which suppressed antimicrobial activity against *S.aureus* 533R4. For the strain designation please see Table S1.

Phylogenetic class	Strain code genus A	Phylogenetic class	Strain code genus B
Actinobacteria	AD107	gamma-proteobacteria	AD14
Actinobacteria	AD107	beta-proteobacteria	AD24
Actinobacteria	AD107	gamma-proteobacteria	AD36
Actinobacteria	AD107	beta-proteobacteria	AD62
Actinobacteria	AD107	beta-proteobacteria	AD72
Actinobacteria	AD107	beta-proteobacteria	AD32
Actinobacteria	AD107	Bacilli	AD83
Actinobacteria	AD107	beta-proteobacteria	AD99
Actinobacteria	AD107	gamma-proteobacteria	AD100
Actinobacteria	AD107	alpha-proteobacteria	AD140
Actinobacteria	AD107	Actinobacteria	AD22
Actinobacteria	AD107	Bacilli	AD87
Actinobacteria	AD107	beta-proteobacteria	AD103
Actinobacteria	AD107	beta-proteobacteria	AD127
Actinobacteria	AD107	Flavobacteria	AD146
Flavobacteria	AD41	beta-proteobacteria	AD19
Flavobacteria	AD41	beta-proteobacteria	AD67
Flavobacteria	AD41	beta-proteobacteria	AD25
Flavobacteria	AD41	Actinobacteria	AD94
Flavobacteria	AD41	beta-proteobacteria	AD96
Flavobacteria	AD41	beta-proteobacteria	AD145
Flavobacteria	AD41	Flavobacteria	AD151
Flavobacteria	AD41	gamma-proteobacteria	AD16
Flavobacteria	AD41	beta-proteobacteria	AD54
Flavobacteria	AD41	gamma-proteobacteria	AD8
Flavobacteria	AD41	beta-proteobacteria	AD68
Flavobacteria	AD41	beta-proteobacteria	AD32
Flavobacteria	AD41	beta-proteobacteria	AD58
Flavobacteria	AD41	gamma-proteobacteria	AD5
Flavobacteria	AD41	gamma-proteobacteria	AD79
Flavobacteria	AD41	Bacilli	AD83
Flavobacteria	AD41	Flavobacteria	AD134
Flavobacteria	AD41	Flavobacteria	AD142
Flavobacteria	AD41	beta-proteobacteria	AD24
Flavobacteria	AD41	Bacilli	AD93
Flavobacteria	AD41	beta-proteobacteria	AD27
Bacilli	AD87	gamma-proteobacteria	AD16
Bacilli	AD87	Actinobacteria	AD22
Bacilli	AD87	alpha-proteobacteria	AD38
Bacilli	AD87	Flavobacteria	AD44
Bacilli	AD87	beta-proteobacteria	AD64
Bacilli	AD87	beta-proteobacteria	AD70
Bacilli	AD87	gamma-proteobacteria	AD20
Bacilli	AD87	beta-proteobacteria	AD26
Bacilli	AD87	beta-proteobacteria	AD98
Bacilli	AD87	Bacilli	AD106
Bacilli	AD87	Flavobacteria	AD142
Bacilli	AD87	Flavobacteria	AD149
Bacilli	AD87	gamma-proteobacteria	AD21
Bacilli	AD87	Flavobacteria	AD48
Bacilli	AD87	alpha-proteobacteria	AD148
Bacilli	AD87	Flavobacteria	AD155
Bacilli	AD87	beta-proteobacteria	AD10
Bacilli	AD87	beta-proteobacteria	AD95
Bacilli	AD87	beta-proteobacteria	AD65
Bacilli	AD87	beta-proteobacteria	AD71
Bacilli	AD87	gamma-proteobacteria	AD14
Bacilli	AD87	gamma-proteobacteria	AD36
Bacilli	AD87	beta-proteobacteria	AD62
Bacilli	AD87	beta-proteobacteria	AD89
Bacilli	AD87	Bacilli	AD83
Bacilli	AD87	beta-proteobacteria	AD101

Table S10 continuation

Phylogenetic class	Strain code genus A	Phylogenetic class	Strain code genus B
Bacilli	AD87	gamma-proteobacteria	AD100
Bacilli	AD87	alpha-proteobacteria	AD140
Bacilli	AD87	beta-proteobacteria	AD144
Bacilli	AD87	Actinobacteria	AD110
Bacilli	AD87	Bacilli	AD50
Bacilli	AD87	Actinobacteria	AD107
Bacilli	AD93	beta-proteobacteria	AD18
Bacilli	AD93	gamma-proteobacteria	AD40
Bacilli	AD93	beta-proteobacteria	AD66
Bacilli	AD93	beta-proteobacteria	AD19
Bacilli	AD93	gamma-proteobacteria	AD114
Bacilli	AD93	beta-proteobacteria	AD144
Bacilli	AD93	alpha-proteobacteria	AD152
Bacilli	AD93	gamma-proteobacteria	AD17
Bacilli	AD93	gamma-proteobacteria	AD52
Bacilli	AD93	beta-proteobacteria	AD67
Bacilli	AD93	Flavobacteria	AD41
Bacilli	AD93	beta-proteobacteria	AD25
Bacilli	AD93	Actinobacteria	AD92
Bacilli	AD93	Actinobacteria	AD94
Bacilli	AD93	beta-proteobacteria	AD96
Bacilli	AD93	beta-proteobacteria	AD145
Bacilli	AD93	Flavobacteria	AD151
Bacilli	AD93	gamma-proteobacteria	AD16
Bacilli	AD93	beta-proteobacteria	AD119
Bacilli	AD93	beta-proteobacteria	AD7
alpha-proteobacteria	AD113	gamma-proteobacteria	AD17
alpha-proteobacteria	AD113	beta-proteobacteria	AD39
alpha-proteobacteria	AD113	beta-proteobacteria	AD65
alpha-proteobacteria	AD113	gamma-proteobacteria	AD21
alpha-proteobacteria	AD113	Actinobacteria	AD92
alpha-proteobacteria	AD113	beta-proteobacteria	AD143
alpha-proteobacteria	AD113	alpha-proteobacteria	AD153
alpha-proteobacteria	AD113	beta-proteobacteria	AD19
alpha-proteobacteria	AD113	alpha-proteobacteria	AD51
alpha-proteobacteria	AD113	beta-proteobacteria	AD66
alpha-proteobacteria	AD113	Flavobacteria	AD91
alpha-proteobacteria	AD113	Flavobacteria	AD146
alpha-proteobacteria	AD113	beta-proteobacteria	AD118
gamma-proteobacteria	AD4	beta-proteobacteria	AD28
gamma-proteobacteria	AD4	gamma-proteobacteria	AD52
gamma-proteobacteria	AD4	gamma-proteobacteria	AD8
gamma-proteobacteria	AD4	gamma-proteobacteria	AD5
gamma-proteobacteria	AD4	beta-proteobacteria	AD76
gamma-proteobacteria	AD4	gamma-proteobacteria	AD104
gamma-proteobacteria	AD4	Flavobacteria	AD131
gamma-proteobacteria	AD4	alpha-proteobacteria	AD140
gamma-proteobacteria	AD4	beta-proteobacteria	AD30
gamma-proteobacteria	AD4	beta-proteobacteria	AD26
gamma-proteobacteria	AD4	Actinobacteria	AD29
gamma-proteobacteria	AD4	alpha-proteobacteria	AD51
gamma-proteobacteria	AD4	beta-proteobacteria	AD54
gamma-proteobacteria	AD4	beta-proteobacteria	AD74
gamma-proteobacteria	AD4	beta-proteobacteria	AD77
gamma-proteobacteria	AD4	gamma-proteobacteria	AD105
gamma-proteobacteria	AD4	alpha-proteobacteria	AD132
gamma-proteobacteria	AD4	beta-proteobacteria	AD145
gamma-proteobacteria	AD4	beta-proteobacteria	AD39
gamma-proteobacteria	AD4	beta-proteobacteria	AD009
gamma-proteobacteria	AD4	beta-proteobacteria	AD103
gamma-proteobacteria	AD4	gamma-proteobacteria	AD120

Table S10 continuation

Phylogenetic class	Strain code genus A	Phylogenetic class	Strain code genus B
gamma-proteobacteria	AD4	alpha-proteobacteria	AD153
gamma-proteobacteria	AD4	beta-proteobacteria	AD137
gamma-proteobacteria	AD4	beta-proteobacteria	AD64
gamma-proteobacteria	AD4	alpha-proteobacteria	AD34
gamma-proteobacteria	AD4	beta-proteobacteria	AD37
gamma-proteobacteria	AD4	Bacilli	AD78
gamma-proteobacteria	AD4	gamma-proteobacteria	AD2
gamma-proteobacteria	AD4	beta-proteobacteria	AD3
gamma-proteobacteria	AD5	Actinobacteria	AD29
gamma-proteobacteria	AD5	beta-proteobacteria	AD54
gamma-proteobacteria	AD5	gamma-proteobacteria	AD4
gamma-proteobacteria	AD5	gamma-proteobacteria	AD8
gamma-proteobacteria	AD5	beta-proteobacteria	AD77
gamma-proteobacteria	AD5	gamma-proteobacteria	AD105
gamma-proteobacteria	AD5	alpha-proteobacteria	AD132
gamma-proteobacteria	AD5	beta-proteobacteria	AD145
gamma-proteobacteria	AD5	beta-proteobacteria	AD25
gamma-proteobacteria	AD5	beta-proteobacteria	AD009
gamma-proteobacteria	AD5	beta-proteobacteria	AD32
gamma-proteobacteria	AD5	Flavobacteria	AD48
gamma-proteobacteria	AD5	beta-proteobacteria	AD58
gamma-proteobacteria	AD5	beta-proteobacteria	AD73
gamma-proteobacteria	AD5	gamma-proteobacteria	AD79
gamma-proteobacteria	AD5	Bacilli	AD83
gamma-proteobacteria	AD5	Flavobacteria	AD134
gamma-proteobacteria	AD5	Flavobacteria	AD142
gamma-proteobacteria	AD5	beta-proteobacteria	AD37
gamma-proteobacteria	AD5	beta-proteobacteria	AD27
gamma-proteobacteria	AD5	beta-proteobacteria	AD24
gamma-proteobacteria	AD5	beta-proteobacteria	AD102
gamma-proteobacteria	AD5	Bacilli	AD116
gamma-proteobacteria	AD5	alpha-proteobacteria	AD152
gamma-proteobacteria	AD5	alpha-proteobacteria	AD136
gamma-proteobacteria	AD5	beta-proteobacteria	AD63
gamma-proteobacteria	AD5	Flavobacteria	AD41
gamma-proteobacteria	AD5	Bacilli	AD78
gamma-proteobacteria	AD5	beta-proteobacteria	AD81
gamma-proteobacteria	AD5	gamma-proteobacteria	AD104
gamma-proteobacteria	AD5	beta-proteobacteria	AD75
gamma-proteobacteria	AD5	beta-proteobacteria	AD3
gamma-proteobacteria	AD5	Actinobacteria	AD12
gamma-proteobacteria	AD5	Actinobacteria	AD108
gamma-proteobacteria	AD6	beta-proteobacteria	AD30
gamma-proteobacteria	AD6	gamma-proteobacteria	AD56
gamma-proteobacteria	AD6	Bacilli	AD78
gamma-proteobacteria	AD6	beta-proteobacteria	AD81
gamma-proteobacteria	AD6	beta-proteobacteria	AD133
gamma-proteobacteria	AD6	Actinobacteria	AD141
gamma-proteobacteria	AD6	beta-proteobacteria	AD28
gamma-proteobacteria	AD6	beta-proteobacteria	AD24
gamma-proteobacteria	AD6	beta-proteobacteria	AD11
gamma-proteobacteria	AD6	beta-proteobacteria	AD30
gamma-proteobacteria	AD6	gamma-proteobacteria	AD46
gamma-proteobacteria	AD6	beta-proteobacteria	AD72
gamma-proteobacteria	AD6	beta-proteobacteria	AD32
gamma-proteobacteria	AD6	beta-proteobacteria	AD99
gamma-proteobacteria	AD6	beta-proteobacteria	AD97
gamma-proteobacteria	AD6	Flavobacteria	AD151
gamma-proteobacteria	AD6	alpha-proteobacteria	AD140
gamma-proteobacteria	AD6	beta-proteobacteria	AD62
gamma-proteobacteria	AD6	beta-proteobacteria	AD39
gamma-proteobacteria	AD6	beta-proteobacteria	AD80

Table S10 continuation

Phylogenetic class	Strain code genus A	Phylogenetic class	Strain code genus B
gamma-proteobacteria	AD6	beta-proteobacteria	AD103
gamma-proteobacteria	AD6	beta-proteobacteria	AD73
gamma-proteobacteria	AD6	beta-proteobacteria	AD7
gamma-proteobacteria	AD8	beta-proteobacteria	AD32
gamma-proteobacteria	AD8	beta-proteobacteria	AD58
gamma-proteobacteria	AD8	gamma-proteobacteria	AD4
gamma-proteobacteria	AD8	gamma-proteobacteria	AD5
gamma-proteobacteria	AD8	gamma-proteobacteria	AD79
gamma-proteobacteria	AD8	Bacilli	AD83
gamma-proteobacteria	AD8	Flavobacteria	AD134
gamma-proteobacteria	AD8	Flavobacteria	AD142
gamma-proteobacteria	AD8	beta-proteobacteria	AD27
gamma-proteobacteria	AD8	beta-proteobacteria	AD24
gamma-proteobacteria	AD8	beta-proteobacteria	AD28
gamma-proteobacteria	AD8	gamma-proteobacteria	AD52
gamma-proteobacteria	AD8	beta-proteobacteria	AD76
gamma-proteobacteria	AD8	gamma-proteobacteria	AD104
gamma-proteobacteria	AD8	Flavobacteria	AD131
gamma-proteobacteria	AD8	alpha-proteobacteria	AD140
gamma-proteobacteria	AD8	beta-proteobacteria	AD30
gamma-proteobacteria	AD8	Flavobacteria	AD41
gamma-proteobacteria	AD8	alpha-proteobacteria	AD34
gamma-proteobacteria	AD8	beta-proteobacteria	AD81
gamma-proteobacteria	AD8	beta-proteobacteria	AD77
gamma-proteobacteria	AD8	Actinobacteria	AD12
gamma-proteobacteria	AD8	gamma-proteobacteria	AD2
beta-proteobacteria	AD9	beta-proteobacteria	AD33
beta-proteobacteria	AD9	beta-proteobacteria	AD59
beta-proteobacteria	AD9	beta-proteobacteria	AD10
beta-proteobacteria	AD9	beta-proteobacteria	AD80
beta-proteobacteria	AD9	beta-proteobacteria	AD88
beta-proteobacteria	AD9	alpha-proteobacteria	AD136
beta-proteobacteria	AD9	Flavobacteria	AD91
beta-proteobacteria	AD9	beta-proteobacteria	AD26
beta-proteobacteria	AD9	beta-proteobacteria	AD32
beta-proteobacteria	AD9	alpha-proteobacteria	AD34
beta-proteobacteria	AD9	beta-proteobacteria	AD60
beta-proteobacteria	AD9	beta-proteobacteria	AD81
beta-proteobacteria	AD9	beta-proteobacteria	AD89
beta-proteobacteria	AD9	beta-proteobacteria	AD137
beta-proteobacteria	AD9	gamma-proteobacteria	AD157
beta-proteobacteria	AD9	beta-proteobacteria	AD25
beta-proteobacteria	AD9	Flavobacteria	AD42
beta-proteobacteria	AD9	Flavobacteria	AD43
beta-proteobacteria	AD9	beta-proteobacteria	AD82
beta-proteobacteria	AD9	Bacilli	AD83
beta-proteobacteria	AD9	Actinobacteria	AD31
beta-proteobacteria	AD9	Bacilli	AD50
gamma-proteobacteria	AD14	gamma-proteobacteria	AD36
gamma-proteobacteria	AD14	beta-proteobacteria	AD62
gamma-proteobacteria	AD14	Bacilli	AD83
gamma-proteobacteria	AD14	gamma-proteobacteria	AD100
gamma-proteobacteria	AD14	alpha-proteobacteria	AD140
gamma-proteobacteria	AD14	beta-proteobacteria	AD144
gamma-proteobacteria	AD14	Actinobacteria	AD22
gamma-proteobacteria	AD14	gamma-proteobacteria	AD20
gamma-proteobacteria	AD14	Flavobacteria	AD44
gamma-proteobacteria	AD14	Flavobacteria	AD42
gamma-proteobacteria	AD14	beta-proteobacteria	AD68
gamma-proteobacteria	AD14	beta-proteobacteria	AD24
gamma-proteobacteria	AD14	beta-proteobacteria	AD95

Table S10 continuation

Phylogenetic class	Strain code genus A	Phylogenetic class	Strain code genus B
gamma-proteobacteria	AD14	Bacilli	AD117
gamma-proteobacteria	AD14	Flavobacteria	AD146
gamma-proteobacteria	AD14	alpha-proteobacteria	AD159
gamma-proteobacteria	AD14	gamma-proteobacteria	AD56
gamma-proteobacteria	AD14	Bacilli	AD87
gamma-proteobacteria	AD14	beta-proteobacteria	AD98
gamma-proteobacteria	AD14	beta-proteobacteria	AD69
gamma-proteobacteria	AD14	Actinobacteria	AD107
gamma-proteobacteria	AD14	Actinobacteria	AD12
gamma-proteobacteria	AD16	alpha-proteobacteria	AD38
gamma-proteobacteria	AD16	beta-proteobacteria	AD64
gamma-proteobacteria	AD16	gamma-proteobacteria	AD20
gamma-proteobacteria	AD16	Bacilli	AD87
gamma-proteobacteria	AD16	Bacilli	AD106
gamma-proteobacteria	AD16	Flavobacteria	AD142
gamma-proteobacteria	AD16	Flavobacteria	AD149
gamma-proteobacteria	AD16	gamma-proteobacteria	AD21
gamma-proteobacteria	AD16	beta-proteobacteria	AD19
gamma-proteobacteria	AD16	Flavobacteria	AD48
gamma-proteobacteria	AD16	Flavobacteria	AD41
gamma-proteobacteria	AD16	beta-proteobacteria	AD73
gamma-proteobacteria	AD16	beta-proteobacteria	AD67
gamma-proteobacteria	AD16	beta-proteobacteria	AD25
gamma-proteobacteria	AD16	Actinobacteria	AD94
gamma-proteobacteria	AD16	beta-proteobacteria	AD96
gamma-proteobacteria	AD16	beta-proteobacteria	AD145
gamma-proteobacteria	AD16	Flavobacteria	AD151
gamma-proteobacteria	AD16	beta-proteobacteria	AD54
gamma-proteobacteria	AD16	beta-proteobacteria	AD65
gamma-proteobacteria	AD16	beta-proteobacteria	AD89
gamma-proteobacteria	AD16	Bacilli	AD93
gamma-proteobacteria	AD16	beta-proteobacteria	AD68
gamma-proteobacteria	AD16	Actinobacteria	AD110
gamma-proteobacteria	AD16	beta-proteobacteria	AD7
gamma-proteobacteria	AD17	beta-proteobacteria	AD39
gamma-proteobacteria	AD17	beta-proteobacteria	AD65
gamma-proteobacteria	AD17	gamma-proteobacteria	AD21
gamma-proteobacteria	AD17	Actinobacteria	AD92
gamma-proteobacteria	AD17	alpha-proteobacteria	AD113
gamma-proteobacteria	AD17	beta-proteobacteria	AD143
gamma-proteobacteria	AD17	alpha-proteobacteria	AD153
gamma-proteobacteria	AD17	beta-proteobacteria	AD19
gamma-proteobacteria	AD17	beta-proteobacteria	AD18
gamma-proteobacteria	AD17	alpha-proteobacteria	AD51
gamma-proteobacteria	AD17	gamma-proteobacteria	AD40
gamma-proteobacteria	AD17	beta-proteobacteria	AD66
gamma-proteobacteria	AD17	beta-proteobacteria	AD19
gamma-proteobacteria	AD17	Bacilli	AD93
gamma-proteobacteria	AD17	gamma-proteobacteria	AD114
gamma-proteobacteria	AD17	beta-proteobacteria	AD144
gamma-proteobacteria	AD17	alpha-proteobacteria	AD152
gamma-proteobacteria	AD17	gamma-proteobacteria	AD52
gamma-proteobacteria	AD17	Flavobacteria	AD91
gamma-proteobacteria	AD17	beta-proteobacteria	AD67
gamma-proteobacteria	AD17	beta-proteobacteria	AD118
gamma-proteobacteria	AD17	beta-proteobacteria	AD119
beta-proteobacteria	AD19	Flavobacteria	AD41
beta-proteobacteria	AD19	beta-proteobacteria	AD67
beta-proteobacteria	AD19	beta-proteobacteria	AD18
beta-proteobacteria	AD19	beta-proteobacteria	AD25
beta-proteobacteria	AD19	Actinobacteria	AD94
beta-proteobacteria	AD19	beta-proteobacteria	AD96

Table S10 continuation

Phylogenetic class	Strain code genus A	Phylogenetic class	Strain code genus B
beta-proteobacteria	AD19	beta-proteobacteria	AD145
beta-proteobacteria	AD19	Flavobacteria	AD151
beta-proteobacteria	AD19	gamma-proteobacteria	AD16
beta-proteobacteria	AD19	gamma-proteobacteria	AD17
beta-proteobacteria	AD19	beta-proteobacteria	AD54
beta-proteobacteria	AD19	gamma-proteobacteria	AD40
beta-proteobacteria	AD19	beta-proteobacteria	AD39
beta-proteobacteria	AD19	beta-proteobacteria	AD66
beta-proteobacteria	AD19	beta-proteobacteria	AD65
beta-proteobacteria	AD19	Bacilli	AD93
beta-proteobacteria	AD19	gamma-proteobacteria	AD114
beta-proteobacteria	AD19	beta-proteobacteria	AD144
beta-proteobacteria	AD19	alpha-proteobacteria	AD152
beta-proteobacteria	AD19	gamma-proteobacteria	AD21
beta-proteobacteria	AD19	gamma-proteobacteria	AD52
beta-proteobacteria	AD19	Actinobacteria	AD92
beta-proteobacteria	AD19	alpha-proteobacteria	AD113
beta-proteobacteria	AD19	beta-proteobacteria	AD143
beta-proteobacteria	AD19	alpha-proteobacteria	AD153
beta-proteobacteria	AD19	alpha-proteobacteria	AD51
beta-proteobacteria	AD19	beta-proteobacteria	AD68
beta-proteobacteria	AD19	Flavobacteria	AD91
beta-proteobacteria	AD19	beta-proteobacteria	AD7
beta-proteobacteria	AD19	beta-proteobacteria	AD119
beta-proteobacteria	AD19	beta-proteobacteria	AD118
beta-proteobacteria	AD35	beta-proteobacteria	AD11
beta-proteobacteria	AD35	beta-proteobacteria	AD61
beta-proteobacteria	AD35	beta-proteobacteria	AD15
beta-proteobacteria	AD35	beta-proteobacteria	AD82
beta-proteobacteria	AD35	beta-proteobacteria	AD98
beta-proteobacteria	AD35	beta-proteobacteria	AD138
beta-proteobacteria	AD35	beta-proteobacteria	AD143
beta-proteobacteria	AD35	beta-proteobacteria	AD23
beta-proteobacteria	AD35	beta-proteobacteria	AD61
beta-proteobacteria	AD35	Flavobacteria	AD45
beta-proteobacteria	AD35	Flavobacteria	AD86
beta-proteobacteria	AD35	beta-proteobacteria	AD55
beta-proteobacteria	AD39	gamma-proteobacteria	AD17
beta-proteobacteria	AD39	beta-proteobacteria	AD26
beta-proteobacteria	AD39	beta-proteobacteria	AD65
beta-proteobacteria	AD39	gamma-proteobacteria	AD21
beta-proteobacteria	AD39	alpha-proteobacteria	AD51
beta-proteobacteria	AD39	Actinobacteria	AD92
beta-proteobacteria	AD39	alpha-proteobacteria	AD113
beta-proteobacteria	AD39	beta-proteobacteria	AD143
beta-proteobacteria	AD39	alpha-proteobacteria	AD153
beta-proteobacteria	AD39	beta-proteobacteria	AD19
beta-proteobacteria	AD39	alpha-proteobacteria	AD51
beta-proteobacteria	AD39	beta-proteobacteria	AD74
beta-proteobacteria	AD39	beta-proteobacteria	AD103
beta-proteobacteria	AD39	gamma-proteobacteria	AD120
beta-proteobacteria	AD39	beta-proteobacteria	AD137
beta-proteobacteria	AD39	gamma-proteobacteria	AD4
beta-proteobacteria	AD39	beta-proteobacteria	AD64
beta-proteobacteria	AD39	gamma-proteobacteria	AD6
beta-proteobacteria	AD39	beta-proteobacteria	AD66
beta-proteobacteria	AD39	beta-proteobacteria	AD30
beta-proteobacteria	AD39	gamma-proteobacteria	AD56
beta-proteobacteria	AD39	beta-proteobacteria	AD76
beta-proteobacteria	AD39	Bacilli	AD78
beta-proteobacteria	AD39	beta-proteobacteria	AD81
beta-proteobacteria	AD39	beta-proteobacteria	AD133

Table S10 continuation

Phylogenetic class	Strain code genus A	Phylogenetic class	Strain code genus B
beta-proteobacteria	AD39	Actinobacteria	AD141
beta-proteobacteria	AD39	beta-proteobacteria	AD11
beta-proteobacteria	AD39	Flavobacteria	AD91
beta-proteobacteria	AD39	gamma-proteobacteria	AD105
beta-proteobacteria	AD39	beta-proteobacteria	AD28
beta-proteobacteria	AD39	beta-proteobacteria	AD118
beta-proteobacteria	AD39	Actinobacteria	AD110
beta-proteobacteria	AD54	gamma-proteobacteria	AD5
beta-proteobacteria	AD54	beta-proteobacteria	AD19
beta-proteobacteria	AD54	Actinobacteria	AD29
beta-proteobacteria	AD54	Flavobacteria	AD41
beta-proteobacteria	AD54	gamma-proteobacteria	AD4
beta-proteobacteria	AD54	beta-proteobacteria	AD77
beta-proteobacteria	AD54	gamma-proteobacteria	AD105
beta-proteobacteria	AD54	alpha-proteobacteria	AD132
beta-proteobacteria	AD54	beta-proteobacteria	AD145
beta-proteobacteria	AD54	beta-proteobacteria	AD67
beta-proteobacteria	AD54	beta-proteobacteria	AD009
beta-proteobacteria	AD54	beta-proteobacteria	AD25
beta-proteobacteria	AD54	Actinobacteria	AD94
beta-proteobacteria	AD54	beta-proteobacteria	AD96
beta-proteobacteria	AD54	Flavobacteria	AD151
beta-proteobacteria	AD54	gamma-proteobacteria	AD16
beta-proteobacteria	AD54	beta-proteobacteria	AD37
beta-proteobacteria	AD54	beta-proteobacteria	AD68
beta-proteobacteria	AD54	Bacilli	AD78
beta-proteobacteria	AD54	beta-proteobacteria	AD3
beta-proteobacteria	AD54	beta-proteobacteria	AD7
beta-proteobacteria	AD64	gamma-proteobacteria	AD16
beta-proteobacteria	AD64	beta-proteobacteria	AD26
beta-proteobacteria	AD64	alpha-proteobacteria	AD38
beta-proteobacteria	AD64	alpha-proteobacteria	AD51
beta-proteobacteria	AD64	gamma-proteobacteria	AD20
beta-proteobacteria	AD64	Bacilli	AD87
beta-proteobacteria	AD64	Bacilli	AD106
beta-proteobacteria	AD64	Flavobacteria	AD142
beta-proteobacteria	AD64	Flavobacteria	AD149
beta-proteobacteria	AD64	gamma-proteobacteria	AD21
beta-proteobacteria	AD64	beta-proteobacteria	AD74
beta-proteobacteria	AD64	Flavobacteria	AD48
beta-proteobacteria	AD64	beta-proteobacteria	AD39
beta-proteobacteria	AD64	beta-proteobacteria	AD103
beta-proteobacteria	AD64	gamma-proteobacteria	AD120
beta-proteobacteria	AD64	alpha-proteobacteria	AD153
beta-proteobacteria	AD64	beta-proteobacteria	AD137
beta-proteobacteria	AD64	gamma-proteobacteria	AD4
beta-proteobacteria	AD64	beta-proteobacteria	AD65
beta-proteobacteria	AD64	beta-proteobacteria	AD76
beta-proteobacteria	AD64	beta-proteobacteria	AD89
beta-proteobacteria	AD64	beta-proteobacteria	AD123
beta-proteobacteria	AD64	Actinobacteria	AD110
beta-proteobacteria	AD66	beta-proteobacteria	AD18
beta-proteobacteria	AD66	gamma-proteobacteria	AD40
beta-proteobacteria	AD66	beta-proteobacteria	AD19
beta-proteobacteria	AD66	Bacilli	AD93
beta-proteobacteria	AD66	gamma-proteobacteria	AD114
beta-proteobacteria	AD66	beta-proteobacteria	AD144
beta-proteobacteria	AD66	alpha-proteobacteria	AD152
beta-proteobacteria	AD66	gamma-proteobacteria	AD17
beta-proteobacteria	AD66	gamma-proteobacteria	AD52
beta-proteobacteria	AD66	beta-proteobacteria	AD39
beta-proteobacteria	AD66	beta-proteobacteria	AD67

Table S10 continuation

Phylogenetic class	Strain code genus A	Phylogenetic class	Strain code genus B
beta-proteobacteria	AD66	beta-proteobacteria	AD65
beta-proteobacteria	AD66	gamma-proteobacteria	AD21
beta-proteobacteria	AD66	Actinobacteria	AD92
beta-proteobacteria	AD66	alpha-proteobacteria	AD113
beta-proteobacteria	AD66	beta-proteobacteria	AD143
beta-proteobacteria	AD66	alpha-proteobacteria	AD153
beta-proteobacteria	AD66	alpha-proteobacteria	AD51
beta-proteobacteria	AD66	gamma-proteobacteria	AD125
beta-proteobacteria	AD66	beta-proteobacteria	AD119
gamma-proteobacteria	AD79	gamma-proteobacteria	AD8
gamma-proteobacteria	AD79	beta-proteobacteria	AD32
gamma-proteobacteria	AD79	beta-proteobacteria	AD58
gamma-proteobacteria	AD79	gamma-proteobacteria	AD5
gamma-proteobacteria	AD79	Bacilli	AD83
gamma-proteobacteria	AD79	Flavobacteria	AD134
gamma-proteobacteria	AD79	Flavobacteria	AD142
gamma-proteobacteria	AD79	beta-proteobacteria	AD27
gamma-proteobacteria	AD79	beta-proteobacteria	AD24
gamma-proteobacteria	AD79	Flavobacteria	AD41
gamma-proteobacteria	AD79	beta-proteobacteria	AD81
gamma-proteobacteria	AD79	beta-proteobacteria	AD130
gamma-proteobacteria	AD79	Actinobacteria	AD12
beta-proteobacteria	AD81	beta-proteobacteria	AD10
beta-proteobacteria	AD81	gamma-proteobacteria	AD6
beta-proteobacteria	AD81	alpha-proteobacteria	AD34
beta-proteobacteria	AD81	beta-proteobacteria	AD30
beta-proteobacteria	AD81	beta-proteobacteria	AD60
beta-proteobacteria	AD81	gamma-proteobacteria	AD56
beta-proteobacteria	AD81	beta-proteobacteria	AD9
beta-proteobacteria	AD81	Bacilli	AD78
beta-proteobacteria	AD81	beta-proteobacteria	AD89
beta-proteobacteria	AD81	beta-proteobacteria	AD137
beta-proteobacteria	AD81	gamma-proteobacteria	AD157
beta-proteobacteria	AD81	beta-proteobacteria	AD25
beta-proteobacteria	AD81	beta-proteobacteria	AD33
beta-proteobacteria	AD81	beta-proteobacteria	AD133
beta-proteobacteria	AD81	Actinobacteria	AD141
beta-proteobacteria	AD81	beta-proteobacteria	AD28
beta-proteobacteria	AD81	beta-proteobacteria	AD11
beta-proteobacteria	AD81	Flavobacteria	AD43
beta-proteobacteria	AD81	beta-proteobacteria	AD39
beta-proteobacteria	AD81	beta-proteobacteria	AD89
beta-proteobacteria	AD81	beta-proteobacteria	AD32
beta-proteobacteria	AD81	beta-proteobacteria	AD58
beta-proteobacteria	AD81	gamma-proteobacteria	AD5
beta-proteobacteria	AD81	Bacilli	AD83
beta-proteobacteria	AD81	gamma-proteobacteria	AD79
beta-proteobacteria	AD81	beta-proteobacteria	AD80
beta-proteobacteria	AD81	Flavobacteria	AD134
beta-proteobacteria	AD81	Flavobacteria	AD142
beta-proteobacteria	AD81	beta-proteobacteria	AD27
beta-proteobacteria	AD81	Bacilli	AD50
beta-proteobacteria	AD81	beta-proteobacteria	AD7
beta-proteobacteria	AD81	Actinobacteria	AD12
beta-proteobacteria	AD82	beta-proteobacteria	AD11
beta-proteobacteria	AD82	beta-proteobacteria	AD35
beta-proteobacteria	AD82	beta-proteobacteria	AD61
beta-proteobacteria	AD82	beta-proteobacteria	AD15
beta-proteobacteria	AD82	beta-proteobacteria	AD98
beta-proteobacteria	AD82	beta-proteobacteria	AD138
beta-proteobacteria	AD82	beta-proteobacteria	AD143

Table S10 continuation

Phylogenetic class	Strain code genus A	Phylogenetic class	Strain code genus B
beta-proteobacteria	AD82	beta-proteobacteria	AD23
beta-proteobacteria	AD82	Flavobacteria	AD45
beta-proteobacteria	AD82	beta-proteobacteria	AD9
beta-proteobacteria	AD82	beta-proteobacteria	AD33
beta-proteobacteria	AD82	beta-proteobacteria	AD59
beta-proteobacteria	AD82	beta-proteobacteria	AD10
beta-proteobacteria	AD82	Flavobacteria	AD86
beta-proteobacteria	AD82	beta-proteobacteria	AD80
beta-proteobacteria	AD82	beta-proteobacteria	AD88
beta-proteobacteria	AD82	alpha-proteobacteria	AD136
beta-proteobacteria	AD82	Flavobacteria	AD91
beta-proteobacteria	AD82	beta-proteobacteria	AD26
beta-proteobacteria	AD82	beta-proteobacteria	AD55
beta-proteobacteria	AD82	Actinobacteria	AD31
beta-proteobacteria	AD103	beta-proteobacteria	AD26
beta-proteobacteria	AD103	alpha-proteobacteria	AD51
beta-proteobacteria	AD103	beta-proteobacteria	AD74
beta-proteobacteria	AD103	beta-proteobacteria	AD39
beta-proteobacteria	AD103	gamma-proteobacteria	AD120
beta-proteobacteria	AD103	alpha-proteobacteria	AD153
beta-proteobacteria	AD103	beta-proteobacteria	AD137
beta-proteobacteria	AD103	gamma-proteobacteria	AD4
beta-proteobacteria	AD103	beta-proteobacteria	AD64
beta-proteobacteria	AD103	beta-proteobacteria	AD76
beta-proteobacteria	AD103	beta-proteobacteria	AD24
beta-proteobacteria	AD103	gamma-proteobacteria	AD46
beta-proteobacteria	AD103	beta-proteobacteria	AD72
beta-proteobacteria	AD103	beta-proteobacteria	AD32
beta-proteobacteria	AD103	gamma-proteobacteria	AD105
beta-proteobacteria	AD103	beta-proteobacteria	AD99
beta-proteobacteria	AD103	beta-proteobacteria	AD97
beta-proteobacteria	AD103	Flavobacteria	AD151
beta-proteobacteria	AD103	alpha-proteobacteria	AD140
beta-proteobacteria	AD103	gamma-proteobacteria	AD6
beta-proteobacteria	AD103	Actinobacteria	AD110
beta-proteobacteria	AD103	Actinobacteria	AD107
beta-proteobacteria	AD144	beta-proteobacteria	AD18
beta-proteobacteria	AD144	gamma-proteobacteria	AD14
beta-proteobacteria	AD144	gamma-proteobacteria	AD40
beta-proteobacteria	AD144	gamma-proteobacteria	AD36
beta-proteobacteria	AD144	beta-proteobacteria	AD66
beta-proteobacteria	AD144	beta-proteobacteria	AD62
beta-proteobacteria	AD144	beta-proteobacteria	AD19
beta-proteobacteria	AD144	Bacilli	AD93
beta-proteobacteria	AD144	Bacilli	AD83
beta-proteobacteria	AD144	gamma-proteobacteria	AD114
beta-proteobacteria	AD144	gamma-proteobacteria	AD100
beta-proteobacteria	AD144	alpha-proteobacteria	AD140
beta-proteobacteria	AD144	alpha-proteobacteria	AD152
beta-proteobacteria	AD144	gamma-proteobacteria	AD17
beta-proteobacteria	AD144	gamma-proteobacteria	AD52
beta-proteobacteria	AD144	Actinobacteria	AD22
beta-proteobacteria	AD144	Flavobacteria	AD44
beta-proteobacteria	AD144	beta-proteobacteria	AD67
beta-proteobacteria	AD144	Actinobacteria	AD92
beta-proteobacteria	AD144	Bacilli	AD87
gamma-proteobacteria	AD20	Flavobacteria	AD42
gamma-proteobacteria	AD20	beta-proteobacteria	AD68
gamma-proteobacteria	AD20	gamma-proteobacteria	AD16
gamma-proteobacteria	AD20	beta-proteobacteria	AD24
gamma-proteobacteria	AD20	beta-proteobacteria	AD95

Table S10 continuation

Phylogenetic class	Strain code genus A	Phylogenetic class	Strain code genus B
gamma-proteobacteria	AD20	Bacilli	AD117
gamma-proteobacteria	AD20	Flavobacteria	AD146
gamma-proteobacteria	AD20	alpha-proteobacteria	AD159
gamma-proteobacteria	AD20	gamma-proteobacteria	AD14
gamma-proteobacteria	AD20	beta-proteobacteria	AD15
gamma-proteobacteria	AD20	gamma-proteobacteria	AD56
gamma-proteobacteria	AD20	alpha-proteobacteria	AD38
gamma-proteobacteria	AD20	beta-proteobacteria	AD37
gamma-proteobacteria	AD20	beta-proteobacteria	AD64
gamma-proteobacteria	AD20	beta-proteobacteria	AD74
gamma-proteobacteria	AD20	beta-proteobacteria	AD63
gamma-proteobacteria	AD20	Bacilli	AD87
gamma-proteobacteria	AD20	Bacilli	AD106
gamma-proteobacteria	AD20	Flavobacteria	AD142
gamma-proteobacteria	AD20	Flavobacteria	AD149
gamma-proteobacteria	AD20	beta-proteobacteria	AD27
gamma-proteobacteria	AD20	gamma-proteobacteria	AD21
gamma-proteobacteria	AD20	Flavobacteria	AD48
gamma-proteobacteria	AD20	Flavobacteria	AD84
gamma-proteobacteria	AD20	beta-proteobacteria	AD101
gamma-proteobacteria	AD20	Actinobacteria	AD141
gamma-proteobacteria	AD20	Flavobacteria	AD47
gamma-proteobacteria	AD20	beta-proteobacteria	AD69
gamma-proteobacteria	AD20	beta-proteobacteria	AD65
gamma-proteobacteria	AD20	beta-proteobacteria	AD98
gamma-proteobacteria	AD20	beta-proteobacteria	AD89
gamma-proteobacteria	AD20	beta-proteobacteria	AD88
gamma-proteobacteria	AD20	Actinobacteria	AD12
gamma-proteobacteria	AD20	Actinobacteria	AD110
gamma-proteobacteria	AD20	Actinobacteria	AD108
gamma-proteobacteria	AD40	beta-proteobacteria	AD18
gamma-proteobacteria	AD40	beta-proteobacteria	AD66
gamma-proteobacteria	AD40	beta-proteobacteria	AD19
gamma-proteobacteria	AD40	Bacilli	AD93
gamma-proteobacteria	AD40	gamma-proteobacteria	AD114
gamma-proteobacteria	AD40	beta-proteobacteria	AD144
gamma-proteobacteria	AD40	alpha-proteobacteria	AD152
gamma-proteobacteria	AD40	gamma-proteobacteria	AD17
gamma-proteobacteria	AD40	gamma-proteobacteria	AD52
gamma-proteobacteria	AD40	beta-proteobacteria	AD67
gamma-proteobacteria	AD40	Actinobacteria	AD92
gamma-proteobacteria	AD40	beta-proteobacteria	AD119
gamma-proteobacteria	AD52	gamma-proteobacteria	AD4
gamma-proteobacteria	AD52	beta-proteobacteria	AD18
gamma-proteobacteria	AD52	beta-proteobacteria	AD28
gamma-proteobacteria	AD52	gamma-proteobacteria	AD40
gamma-proteobacteria	AD52	gamma-proteobacteria	AD8
gamma-proteobacteria	AD52	beta-proteobacteria	AD76
gamma-proteobacteria	AD52	gamma-proteobacteria	AD104
gamma-proteobacteria	AD52	Flavobacteria	AD131
gamma-proteobacteria	AD52	alpha-proteobacteria	AD140
gamma-proteobacteria	AD52	beta-proteobacteria	AD30
gamma-proteobacteria	AD52	beta-proteobacteria	AD66
gamma-proteobacteria	AD52	gamma-proteobacteria	AD8
gamma-proteobacteria	AD52	beta-proteobacteria	AD19
gamma-proteobacteria	AD52	Bacilli	AD93
gamma-proteobacteria	AD52	gamma-proteobacteria	AD114
gamma-proteobacteria	AD52	beta-proteobacteria	AD144
gamma-proteobacteria	AD52	alpha-proteobacteria	AD152
gamma-proteobacteria	AD52	gamma-proteobacteria	AD17
gamma-proteobacteria	AD52	alpha-proteobacteria	AD34
gamma-proteobacteria	AD52	beta-proteobacteria	AD67

Table S10 continuation

Phylogenetic class	Strain code genus A	Phylogenetic class	Strain code genus B
gamma-proteobacteria	AD52	beta-proteobacteria	AD77
gamma-proteobacteria	AD52	gamma-proteobacteria	AD2
alpha-proteobacteria	AD51	beta-proteobacteria	AD26
alpha-proteobacteria	AD51	gamma-proteobacteria	AD17
alpha-proteobacteria	AD51	beta-proteobacteria	AD74
alpha-proteobacteria	AD51	beta-proteobacteria	AD39
alpha-proteobacteria	AD51	beta-proteobacteria	AD103
alpha-proteobacteria	AD51	gamma-proteobacteria	AD120
alpha-proteobacteria	AD51	alpha-proteobacteria	AD153
alpha-proteobacteria	AD51	beta-proteobacteria	AD137
alpha-proteobacteria	AD51	gamma-proteobacteria	AD4
alpha-proteobacteria	AD51	beta-proteobacteria	AD39
alpha-proteobacteria	AD51	beta-proteobacteria	AD64
alpha-proteobacteria	AD51	beta-proteobacteria	AD65
alpha-proteobacteria	AD51	gamma-proteobacteria	AD21
alpha-proteobacteria	AD51	Actinobacteria	AD92
alpha-proteobacteria	AD51	alpha-proteobacteria	AD113
alpha-proteobacteria	AD51	beta-proteobacteria	AD143
alpha-proteobacteria	AD51	beta-proteobacteria	AD19
alpha-proteobacteria	AD51	beta-proteobacteria	AD76
alpha-proteobacteria	AD51	beta-proteobacteria	AD66
alpha-proteobacteria	AD51	gamma-proteobacteria	AD105
alpha-proteobacteria	AD51	beta-proteobacteria	AD118
